# Fused Ring Engineering Induced Topology Control in Covalent Organic Frameworks: Unlocking Promoted Photocatalytic H_2_O_2_ Production and Selective Methane Oxidation

**DOI:** 10.1002/anie.1288153

**Published:** 2026-04-13

**Authors:** Lei Wang, Zhipeng Xie, Xinyi Zeng, Hetao Xu, Mingyue Wang, Jie Yang, Chao Lin, Wandong Xing, Zhengxiao Guo, Xiong Chen

**Affiliations:** ^1^ State Key Laboratory of Chemistry for NBC Hazards Protection College of Chemistry Fuzhou University Fuzhou P. R. China; ^2^ State Key Laboratory of Photocatalysis on Energy and Environment and Key Laboratory of Advanced Carbon‐Based Functional Material College of Chemistry Fuzhou University Fuzhou P. R. China; ^3^ Department of Chemistry The University of Hong Kong Hong Kong SAR P. R. China

**Keywords:** covalent organic frameworks, exciton dissociation, photocatalytic H_2_O_2_ production, photocatalytic methane oxidation, topology engineering

## Abstract

Efficient solar‐to‐chemical energy conversion and the selective activation of methane remain grand challenges in artificial photosynthesis. Here, we report the rational design of two covalent organic frameworks (COFs), Phen‐TTA and O‐TTA, in which framework topology and π‐conjugation are regulated by pairing a triazine‐based acceptor with either a rigid 1,10‐phenanthroline or a twisted 2,2′‐bipyridine donor. The results show that the Phen‐TTA, bearing a rigid *kgd‐v* topology, features a narrowed bandgap, reduced exciton binding energy, and accelerated charge‐carrier kinetics relative to O‐TTA with an *hcb* topology. Consequently, Phen‐TTA delivers a high photocatalytic H_2_O_2_ production behavior (21.5 mmol h^−1^ g^−1^ and an apparent quantum yield of 5.45% at 450 nm), placing it among the most active COF‐based photocatalysts ever reported. Notably, Phen‐TTA further enables selective photocatalytic methane oxidation to ethanol (30.1 µmol h^−1^ g^−1^, 365 nm irradiation) in the absence of noble‐metal cocatalysts. Mechanistic investigations indicate that the enhanced framework rigidity promotes sequential one‐electron oxygen reduction to H_2_O_2_, while the sustained H_2_O_2_ supply undergoes photolysis to yield ^•^OH that drive C─H activation. This work establishes topology engineering as an effective strategy to overcome excitonic and charge‐transport limitations in polymeric photocatalysts and demonstrates a rare single‐component organic framework for tandem solar‐driven methane valorization.

## Graphical abstract

By modulating the topological structures of two N‐heterocycle‐based covalent organic frameworks, Phen‐TTA with a *kgd‐v* topology exhibits narrower optical bandgap, lower exciton binding energy, and faster charge‐carrier kinetics compared to O‐TTA with an *hcb* topology. This not only results in remarkable photocatalytic H_2_O_2_ production but also enables the selective photocatalytic methane oxidation to ethanol from in situ–generated H_2_O_2_ for Phen‐TTA.

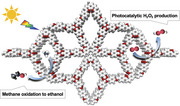

## 1 Introduction

Efficient harnessing of solar energy is imperative to mitigate dwindling fossil resources and escalating environmental concerns [1, 2]. Within the realm of artificial photosynthesis, semiconductor‐mediated photocatalytic hydrogen peroxide (H_2_O_2_) production via two‐electron oxygen reduction has emerged as a compelling pathway for solar‐to‐chemical energy conversion [3, 4, 5]. This approach offers a sustainable alternative to the conventional energy‐intensive anthraquinone process, which relies heavily on hydrogen, noble‐metal catalysts, and organic solvents [6]. Nevertheless, achieving competitive productivity and selectivity in photocatalytic H_2_O_2_ production remains challenging and requires photocatalysts that satisfy several stringent criteria: (i) appropriately aligned band structures or frontier orbital energies to thermodynamically drive both O_2_ reduction and the coupled oxidation half‐reaction, (ii) broad visible‐light absorption to maximize solar harvesting, (iii) efficient exciton dissociation and long‐lived charge separation to suppress recombination, and (iv) well‐defined and selective interfacial active sites that favor H_2_O_2_ formation while minimizing its subsequent decomposition.

In this context, fully organic, metal‐free conjugated polymers have garnered increasing attention owing to their structural tunability, precise synthetic control over optoelectronic properties, and independence from critical raw materials [7, 8, 9, 10, 11, 12]. Among these, covalent organic frameworks (COFs), a fascinating category of crystalline porous polymers assembled from lightweight elements via covalent bonds, stand out due to their modular design, permanent porosity, and remarkable physicochemical stability [13, 14, 15, 16, 17, 18, 19, 20, 21, 22, 23, 24, 25, 26, 27, 28, 29, 30, 31, 32, 33]. Since the seminal report in 2020 by Van Der Voort and co‐workers demonstrating visible‐light‐driven H_2_O_2_ production over two‐dimensional (2D) imine‐linked TAPD‐based COFs [34], this field has experienced rapid growth [35, 36, 37]. Subsequent efforts have explored diverse modification strategies, including linker functionalization [38], molecular doping [39], construction of three‐dimensional (3D) architectures [40, 41], development of nanohybrids [42], heterojunction engineering [43], immobilization of molecular co‐catalysts [44], and post‐synthetic modifications [45, 46, 47, 48], all aimed at enhancing charge separation and catalytic turnover. Despite these advances, rapid charge carrier recombination and inefficient photogenerated charge transport remain persistent bottlenecks, limiting quantum efficiency and underscoring the need for new materials that integrate broad‐spectrum light absorption, efficient charge separation, and optimized redox‐active sites.

**SCHEME 1:**
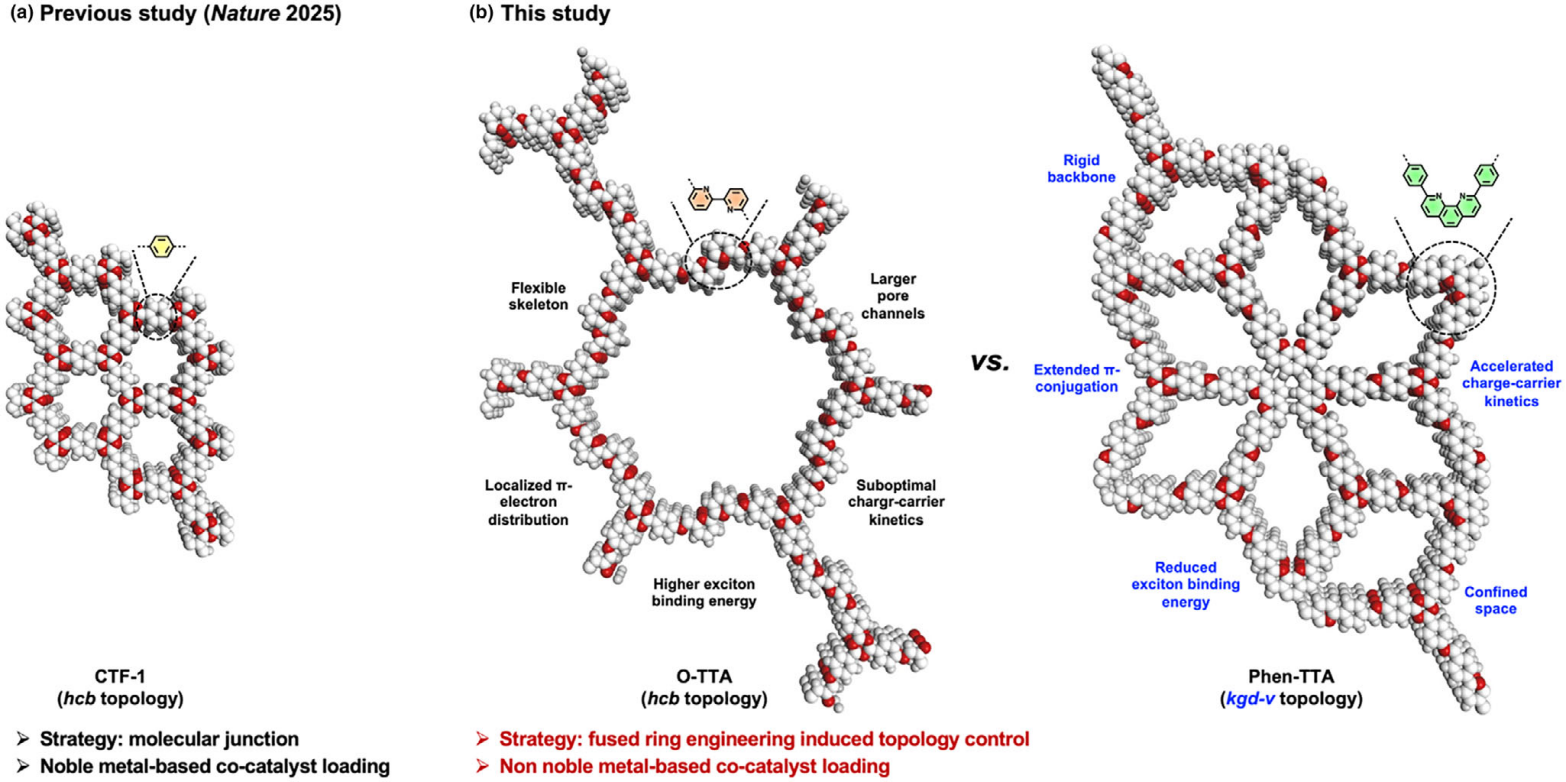
Schematic illustration for the design principles of (a) previous work (CTF‐1) [53] and (b) this study (O‐TTA and Phen‐TTA) for photocatalytic methane oxidation (white: carbon; red: nitrogen; light gray: hydrogen).

Beyond H_2_O_2_ synthesis, the selective oxygenation of methane—the principal component of natural gas—into value‐added oxygenates (e.g., ethanol) under ambient conditions represents one of the most formidable challenges in catalysis, primarily due to the high C─H bond dissociation energy (∼439 kJ mol^−1^) and the scarcity of environmentally benign oxidants capable of enabling selective transformation [49, 50, 51, 52]. In this regard, coupling in situ photocatalytic H_2_O_2_ generation with downstream methane activation offers a compelling route toward a solar‐driven, closed‐loop conversion of CH_4_, O_2_, and H_2_O into liquid oxygenates. To date, however, only a limited number of polymeric photocatalysts (i.e., CTF‐1 (Scheme 1a) and *g*‐C_3_N_4_) have demonstrated such tandem functionality [53], and no COF‐based system has yet been reported to achieve this synergistic transformation, highlighting a significant opportunity.

To this end, herein, we present the rational design and synthesis of two N‐heterocycle‐based COFs, denoted Phen‐TTA and O‐TTA, constructed from a common electron‐deficient 4,4',4''‐(1,3,5‐triazine‐2,4,6‐triyl) trianiline (TTA) core linked with either phenanthroline (Phen) or *o*‐bipyridine (O) as electron‐rich units. These donor moieties were selected to modulate molecular rigidity, π‐conjugation, and framework topology (Scheme 1b). Compared with the twisted 2,2'‐bipyridine motif, the rigidly fused 1,10‐phenanthroline scaffold promotes enhanced π‐electron delocalization and stabilizes the lowest unoccupied molecular orbital (LUMO), resulting in increased framework rigidity and extended π‐conjugation in Phen‐TTA, which adopts a *kgd‐v* topology [16]. Comprehensive experimental and theoretical investigations reveal that Phen‐TTA demonstrates a narrower optical bandgap, facilitated exciton dissociation, more efficient charge transport, thereby delivering superior photocatalytic performance in both H_2_O_2_ production and selective methane oxidation to ethanol. Collectively, these findings establish clear structure–property correlations and provide a design paradigm for the next‐generation COF photocatalysts capable of driving complex, multistep solar‐to‐chemical transformations with high efficiency and selectivity.

## 2 Results and Discussion

### 2.1 Synthesis and Characterizations

**FIGURE 1:**
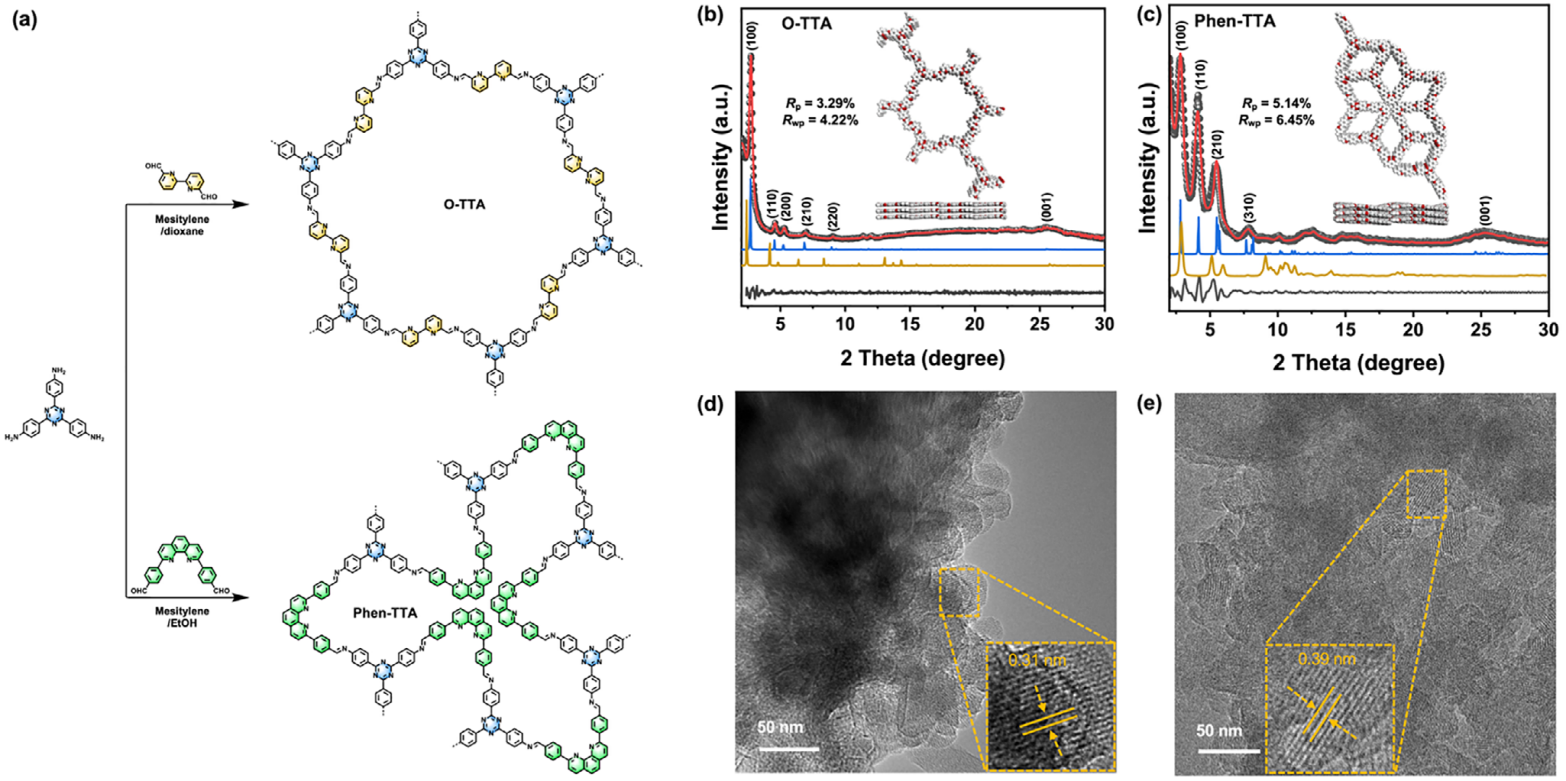
(a) Synthetic routes of O‐TTA and Phen‐TTA. Pawley refinement and experiment PXRD patterns of (b) O‐TTA and (c) Phen‐TTA (Dotted black: experimental; red: refined; blue: simulated AA stacking; orange: simulated AB stacking; black: difference) (Insets: front and side views of the proposed unit cells of AA stacking modes). HR‐TEM images of (d) O‐TTA and (e) Phen‐TTA.

The synthesis of these two COFs, Phen‐TTA and O‐TTA, were conducted via solvothermal condition at 120°C for 3 days (Figure 1a, details see Supporting Information). The successful formation and structural integrity of these COFs were confirmed through comprehensive spectroscopic and structural analyses. Fourier transform infrared (FT‐IR) spectroscopy (Figures S1 and S2) showed a marked attenuation of the C═O stretching bands in the O‐BPy and Phen precursors post‐condensation, accompanied by the emergence of characteristic imine (C═N) stretching bands at 1609 cm^−1^ (O‐TTA) and 1651 cm^−1^ (Phen‐TTA), confirming successful Schiff base condensation. Solid‐state ^13^C NMR spectroscopy (Figures S3 and S4) further validated the structural integrity of the frameworks, with distinct signals at 160.7 ppm (O‐TTA) and 161.5 ppm (Phen‐TTA) attributed to imine carbons, alongside aromatic carbon signals in the 120–140 ppm range. These FT‐IR and ^13^C NMR results collectively verify the incorporation of C═N linkages, affirming their successful synthesis of both COFs. X‐ray photoelectron spectroscopy (XPS) analysis provided additional insights into the surface elemental composition and chemical environments. The XPS survey spectra of O‐TTA and Phen‐TTA (Figure S5a) exhibited characteristic signals for carbon and nitrogen. High‐resolution N 1s spectra of Phen‐TTA (Figure S5b) revealed peaks at 397.5 eV and 398.3 eV, corresponding to pyridine and imine N atoms, respectively. In contrast, O‐TTA displayed higher binding energies for pyridine (398.4 eV) and imine (399.9 eV) nitrogen atoms, attributable to the reduced electron density resulting from the less conjugated *o*‐bipyridine structure compared to the phenanthrene group in Phen‐TTA. These complementary spectroscopic datasets confirm the successful formation of the designed COF architectures.

Powder x‐ray diffraction (PXRD) analysis (Figure 1b,c) revealed high crystallinity for both COFs. O‐TTA (Figure 1b) featured a regular hexagonal pore structure, displaying diffraction peaks at ∼2.7°, 4.6°, 5.3°, 6.9°, 9.0°, and 25.3°, corresponding to the (100), (110), (200), (210), (220), and (001) crystal planes. In contrast, Phen‐TTA (Figure 1c) adopted a *kgd‐v* topology [16], with peaks at ∼2.8°, 4.1°, 5.5°, 7.9°, and 25.2°, assigned to the (100), (110), (210), (310), and (001) planes, respectively. Pawley refinements and structural simulations assuming an eclipsed (AA) stacking mode closely matched the experimental PXRD patterns (Figure 1b,c), confirming that both COFs adopt an AA‐stacked configuration. High‐resolution transmission electron microscopy (HR‐TEM) further visualized ordered crystalline domains, revealing lattice fringes of 0.31 nm and 0.39 nm assigned to the (001) facets of O‐TTA and Phen‐TTA (Figure 1d,e). Scanning electron microscopy (SEM) images (Figure S6) showed that O‐TTA possesses an irregular, block‐like aggregated morphology, whereas Phen‐TTA exhibits a more defined lamellar structure. Nitrogen sorption isotherms collected at 77 K yielded Brunauer–Emmett–Teller (BET) surface areas of 246 m^2^ g^−1^ for O‐TTA and 193 m^2^ g^−1^ for Phen‐TTA, respectively (Figure S7a). Pore size distributions derived from nonlocal density functional theory (NLDFT) indicated that O‐TTA predominantly features mesopores with a characteristic size centered at 2.7 nm. In contrast, Phen‐TTA exhibited a hierarchical pore structure spanning both microporous and mesoporous regimes, with a primary pore size of 1.1 nm and a secondary distribution centered at 3.4 nm (Figure S7b). These results are consistent with the proposed structural models and further substantiate the reliability of the predicted frameworks. Thermogravimetric analysis (TGA) (Figure S8) demonstrated that both COFs exhibited good thermal stability, remaining stable at temperatures above 300°C.

The hydrophilicity of the catalysts was evaluated via water contact angle and water vapor uptake measurements. Contact angle measurements (Figure S9) revealed values of 49.2° for O‐TTA and 28.2° for Phen‐TTA, indicating substantially higher surface wettability for Phen‐TTA. Complementary water vapor uptake isotherms (Figure S10) further confirmed the superior hydrophilicity of Phen‐TTA, showing markedly greater vapor uptake across the measured pressure range. This enhanced hydrophilicity is expected to facilitate more efficient photocatalytic H_2_O_2_ production.

### 2.2 Photophysical and Photo(electro)Chemical Properties

**FIGURE 2:**
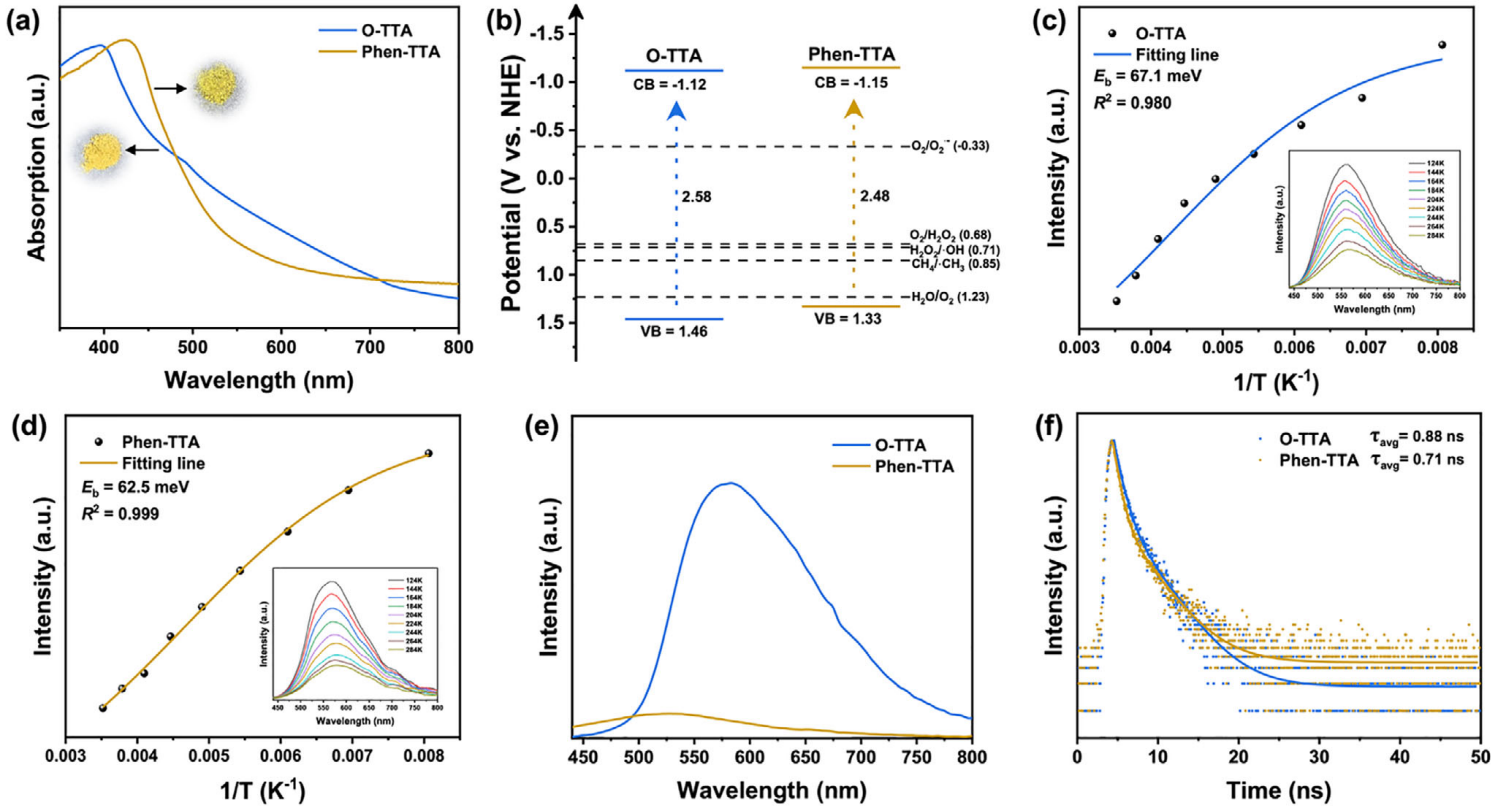
(a) UV/Vis DRS spectra (Insets are the optical images of Phen‐TTA and O‐TTA), (b) Band structures of O‐TTA and Phen‐TTA. Integrated PL emission intensity as a function of temperature from 124 to 284 K for (c) O‐TTA (d) Phen‐TTA (Inset: temperature‐dependent PL spectra). (e) Steady‐state PL spectra (excitation wavelength: 420 nm), (f) Time‐resolution PL decay spectroscopy of O‐TTA and Phen‐TTA.

Ultraviolet‐visible diffuse reflection spectroscopy (UV‐vis DRS) was used to investigate the optical and electronic properties of the two COFs. Both materials exhibited strong light absorption across the visible region (Figure 2a), underscoring their potential for efficient solar energy utilization. Tauc analysis of the UV‐vis DRS (Figure S11) yielded optical band gaps (*E*_g_) of 2.48 eV (Phen‐TTA) and 2.58 eV (O‐TTA), with the narrower band gap of Phen‐TTA indicating enhanced light‐harvesting capability. The band‐edge positions were further determined via Mott–Schottky measurements, which produced flat‐band potentials of −1.32 V (O‐TTA) and −1.35 V (Phen‐TTA) versus Ag/AgCl (Figure S12), confirming their *n*‐type semiconducting character [54]. Combining the flat‐band potentials with the optical band gaps afforded valence band maxima (VBM) of 1.46 V (O‐TTA) and 1.33 V (Phen‐TTA) (Figure 2b). Notably, the conduction band minima (CBM) of both COFs are sufficiently negative relative to the redox potentials required for the direct two‐electron oxygen reduction reaction (2e^−^ ORR, +0.68 V vs. NHE) as well as the stepwise one‐electron ORR (–0.33 V vs. NHE), confirming their thermodynamic suitability for driving photocatalytic O_2_‐to‐H_2_O_2_ reduction.

Temperature‐dependent photoluminescence (PL) spectroscopy (124–284 K) were employed to probe the kinetics of electron‐hole (e^−^–h^+^) pair dissociation in both COFs (Figure 2c,d). Both samples exhibited thermal quenching behavior, and fitting the PL intensities using the Arrhenius equation [55, 56, 57] yielded exciton binding energies (*E*_b_) of 62.5 meV for Phen‐TTA and 67.1 meV for O‐TTA. The lower *E*_b_ of Phen‐TTA indicates facilitated exciton dissociation [58, 59, 60], thereby increasing the population of free charge carriers and likely contributing to higher photocatalytic activity. Steady‐state PL spectra (Figure 2e) further showed noticeably weaker emission for Phen‐TTA than O‐TTA, suggesting suppressed e^−^–h^+^ recombination in the former, attributable to the extended planar conjugation of the phenanthrene moiety. Time‐resolved PL decay profiles (Figure 2f) corroborated these observations, with Phen‐TTA exhibiting a shorter average exciton lifetime (0.71 ns) compared to O‐TTA (0.88 ns), signifying accelerated photogenerated electron migration [61]. Photoelectrochemical measurements provided additional support for these trends, with Phen‐TTA delivering a higher and more stable photocurrent response (Figure S13) and a lower electrochemical impedance (Figure S14) relative to O‐TTA, confirming superior charge separation and transport efficiency [62, 63]. These results collectively highlight the critical influence of molecular‐level design on the photophysical and photoelectrochemical behavior of COF‐based photocatalysts, positioning Phen‐TTA as a highly promising platform for photocatalytic H_2_O_2_ production.

Density functional theory (DFT) calculations provided insights into the electronic structure, revealing HOMO–LUMO gaps of 1.95 eV and 2.33 eV for Phen‐TTA and O‐TTA, respectively (Figure S15). The narrower energy gap of Phen‐TTA affords greater charge transfer opportunities within its extensive π‐conjugated network. In Phen‐TTA, the HOMO is primarily localized on the partial TTA unit and imine bonds, while the LUMO is predominantly distributed on the phenanthrene unit and triazine ring segment (Figure S16), facilitating efficient charge migration. In contrast, O‐TTA exhibits delocalized HOMO and LUMO distributions across its framework (Figure S17), leading to less efficient carrier separation. Evidently, the highly conjugated configuration of the phenanthrene unit in Phen‐TTA markedly increases electron density, enhancing carrier separation and migration capabilities, which underpins its photocatalytic performance.

### 2.3 Photocatalytic H_2_O_2_ Production and Selective Methane Oxidation to Ethanol

Photocatalytic H_2_O_2_ production was first assessed under 420 nm LED irradiation in O_2_‐saturated pure water (20 mL, 10 mg catalyst) (see Supporting Information for details) [64]. Both COFs displayed appreciable activity, with Phen‐TTA generating 29.8 µmol of H_2_O_2_ after 30 min, surpassing O‐TTA's yield of 23.4 µmol under identical conditions. To further enhance performance, a biphasic water/benzyl alcohol (BA) system (18 mL: 2 mL) was employed. In this configuration, the COF photocatalyst primarily dispersed in the organic BA phase, establishing an immiscible liquid–liquid interface. Within the organic phase, photogenerated holes oxidized BA to benzaldehyde, while photogenerated electrons facilitated the oxygen reduction reaction (ORR) at the H_2_O/BA interface to produce H_2_O_2_. Owing to its hydrophilic nature, the generated H_2_O_2_ rapidly diffused into the aqueous phase. This spatially separated biphasic environment not only stabilized the photocatalyst, preventing agglomeration and deactivation, but also enhanced catalytic efficiency by enabling rapid product extraction and continuous benzaldehyde supply.

**FIGURE 3:**
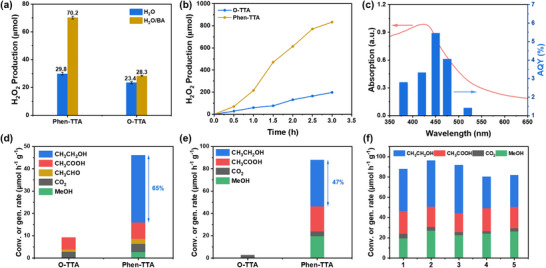
(a) Photocatalytic H_2_O_2_ production activity diagram of Phen‐TTA and O‐TTA with and without sacrificial agent conditions over 0.5 h. (b) Photocatalytic H_2_O_2_ production performance of Phen‐TTA in BA/H_2_O system. (c) Wavelength dependence of AQY on H_2_O_2_ production for Phen‐TTA. Comparison of photocatalytic activity between O‐TTA and Phen‐TTA in methane transformation under (d) 365 nm and (e) 420 nm irradiation. (f) Stability of Phen‐TTA during cyclic photocatalytic methane transformation under 420 nm irradiation.

In the O_2_‐satured water‐BA system, Phen‐TTA (10 mg) achieved a H_2_O_2_ yield of 70.2 µmol after 30 min of irradiation, markedly exceeding its performance in pure water (29.8 µmol) (Figure 3a). Under continuous illumination (Figure 3b), Phen‐TTA delivered a H_2_O_2_ production rate of 215.2 µmol h^−1^, approximately 3.6‐fold higher than that of O‐TTA (60.1 µmol h^−1^). Apparent quantum yield (AQY) measurements (Figure 3c) for Phen‐TTA revealed a wavelength‐dependent trend consistent with UV‐vis DRS results, achieving an AQY of 5.45% at 450 nm, surpassing many previously reported COF‐based photocatalysts (Table S1) [65, 66, 67]. The H_2_O_2_ concentration increased linearly with illumination time, illustrating the steady‐state nature of the photocatalytic process and underscoring the beneficial role of the phenanthrene unit in enhancing activity. Cyclic photocatalytic tests (2‐h cycles) confirmed the durability of Phen‐TTA, with negligible activity loss after four consecutive cycles (Figure S18). Post‐reaction FT‐IR (Figure S19) and PXRD (Figure S20) analyses of recycled Phen‐TTA revealed minimal structural changes, underscoring its robustness and reusability during prolonged photocatalysis.

The multifunctionality of Phen‐TTA and O‐TTA was further demonstrated in the selective oxidation of methane using the in situ–generated H_2_O_2_ as the oxidant under continuous 365 nm irradiation in a flow reactor (5 mg catalyst, 40 sccm CH_4_/O_2_ flow, ambient pressure) (Figure S21, see Supporting Information for details). Phen‐TTA achieved an ethanol production rate of 30.1 µmol h^−1^ g^−1^, with trace acetic acid as a byproduct, whereas O‐TTA produced no detectable ethanol (Figure 3d), consistent with their pronounced difference in H_2_O_2_ generation. A survey of the literature on polymer‐based photocatalytic methane oxidation (Table S2) highlights common challenges, including suboptimal product selectivity and reliance on co‐catalysts. While some polymer catalysts can partially oxidize methane, they typically yield a mixture of oxygenated products, making selective ethanol formation difficult. Additionally, many systems require co‐catalysts or precious metals to achieve appreciable activity, which increases costs and may pose environmental concerns. In contrast, Phen‐TTA exhibits significant advantages. First, it operates as a metal‐free COF without co‐catalysts, reducing both cost and potential environmental impact. Second, it demonstrates high selectivity in ethanol formation from methane oxidation.

To further assess its versatility, methane oxidation was performed under 420 nm LED irradiation, yielding trends consistent with those observed under 365 nm light (Figure 3e), validating the structural and photophysical advantages of Phen‐TTA. Moreover, Phen‐TTA exhibits good stability and reusability, maintaining high catalytic activity over multiple cycles (Figure 3f). Postreaction FT‐IR (Figure S22) and PXRD (Figure S23) analyses of the cycled Phen‐TTA showed minimal structural alteration, underscoring the practical robustness of this COF for sustainable methane valorization.

### 2.4 Mechanistic Insights

To elucidate the mechanism of photocatalytic H_2_O_2_ production by Phen‐TTA, a series of control experiments were conducted (Figure S24). H_2_O_2_ generation decreased markedly under an air atmosphere relative to pure O_2_, was nearly suppressed under Ar, and became negligible in the dark, collectively confirming the essential roles of O_2_ and photoexcitation. Rotating disk electrode (RDE) analysis further revealed average electron transfer numbers (*n*) of 1.70 for O‐TTA and 1.89 for Phen‐TTA (Figure S25), indicating an occurrence for the 2e^−^ ORR pathway, with Phen‐TTA exhibiting higher selectivity due to its electron‐rich phenanthroline unit. Moreover, quenching experiments with AgNO_3_ (e^−^ scavenger) and *p*‐benzoquinone (*p*‐BQ, ^•^O_2_^−^ scavenger) (Figure S26) showed markedly suppression of H_2_O_2_ production, underscoring the critical involvement of photogenerated electrons and ^•^O_2_^−^ as a key intermediate. To further elucidate the ORR pathway during photocatalytic H_2_O_2_ production, isotope‐labeling experiments were conducted using ^18^O_2_ (Figure S27). Upon decomposition of the in situ–generated H_2_O_2_ with MnO_2_, a characteristic signal at *m/z* = 36 (^18^O_2_) is observed, confirming that the oxygen atoms in H_2_O_2_ originate from molecular oxygen. The minor signal at *m/z* = 32 (^16^O_2_) is attributed to slight O_2_ leakage during sample handling. Collectively, these observations suggest that H_2_O_2_ production proceeds via both direct and sequential 2e^−^ ORR pathway, with the sequential 2e^−^ ORR pathway predominating.

**FIGURE 4:**
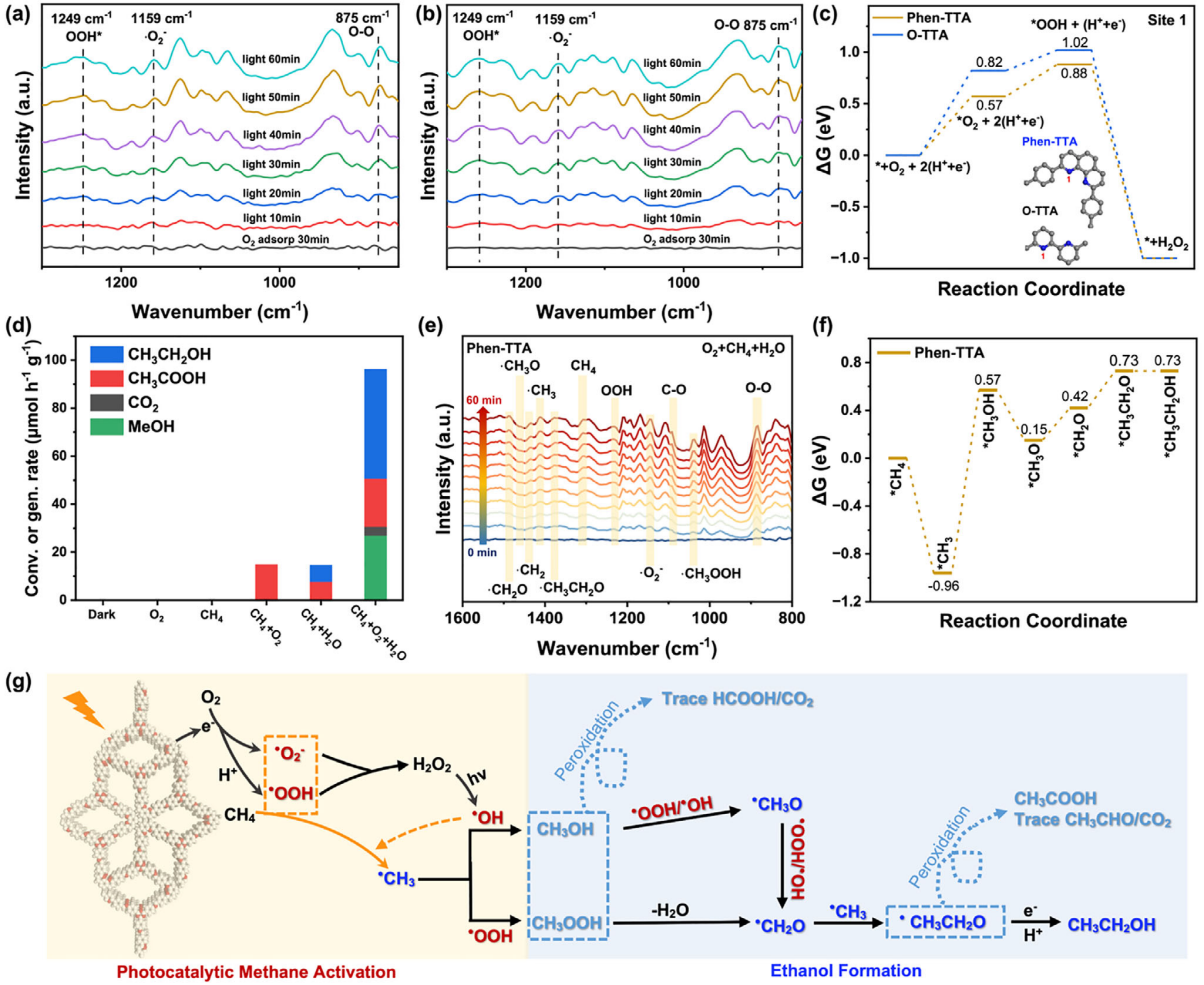
In situ DRIFTS spectra of (a) Phen‐TTA and (b) O‐TTA during H_2_O_2_ production under visible‐light irradiation. (c) DFT calculated Gibbs free energy diagrams of oxygen reduction pathways toward H_2_O_2_ generation on active site 1 of O‐TTA (blue) and Phen‐TTA (orange). (d) Control experiments assessing the photocatalytic methane transformation of Phen‐TTA under 420 nm LED irradiation. (e) In situ DRIFTS spectrum of photocatalytic CH_4_ oxidation over Phen‐TTA during light irradiation (420 nm LED) from 0 to 60 min. (f) DFT calculated free energy diagrams of the CH_4_ conversion reaction pathway on Phen‐TTA. (g) Schematic of the proposed reaction mechanism for photocatalytic CH_4_ oxidation to C_2_H_5_OH.

Electron paramagnetic resonance (EPR) spectroscopy employing 5,5‐dimethyl‐1‐pyrroline N‐oxide (DMPO) as a spin trap in MeOH detected the characteristic six‐line signals of the DMPO‐^•^O_2_^−^ adduct under illumination for both COFs (Figure S28), corroborating the formation of ^•^O_2_^−^ and further supporting a stepwise one‐electron ORR pathway [68]. Additionally, in situ diffuse reflectance infrared Fourier transform spectroscopy (DRIFTS) was used to monitor surface adsorbates and reaction intermediates dynamically during photocatalytic H_2_O_2_ generation. No discernible spectral changes appeared after 30 min of O_2_ adsorption in the dark (Figure 4a,b), ruling out photochromic effects. Under visible light irradiation in a water vapor–saturated O_2_ atmosphere, an O_2_ adsorption band emerged at 875 cm^−1^. With prolonged illumination, vibrational features at 1159 cm^−1^ and 1249 cm^−1^, corresponding to ^•^O_2_^−^ and OOH* intermediates, respectively, intensified progressively. These observations collectively substantiate that both COFs follow a stepwise one‐electron ORR pathway for H_2_O_2_ production.

DFT calculations provided molecular‐level insights into the photocatalytic process. Analysis of potential catalytic sites and their corresponding Gibbs free energies (Figures 4c, and S29–S31) showed that the nitrogen atom on the phenanthroline moiety at site 1 of Phen‐TTA exhibited the lowest Gibbs free energy for *O_2_ formation (*ΔG* = 0.57 eV) among all evaluated sites (site 2: 0.61 eV; site 3: 0.58 eV; site 4: 0.62 eV; site 5: 0.67 eV). In contrast, O‐TTA displayed a higher minimum *ΔG* of 0.69 eV for *O_2_ formation at site 2. Further analysis of the influence of different active sites on proton adsorption capacity revealed that the center hosting the imine bond in Phen‐TTA exhibited the lowest proton adsorption energy (*ΔG* = 0.83 eV), significantly lower than the minimum adsorption energy at site 2 in O‐TTA (*ΔG* = 0.98 eV). This markedly reduced the energy barrier for the rate‐determining step of proton adsorption. The substantially lower *ΔG* values of Phen‐TTA implied a reduced energy barrier for O_2_ activation and subsequent H_2_O_2_ formation, attributable to its extended π‐conjugation and enhanced electron density, which collectively facilitate charge transport and stabilize ORR intermediates.

By integrating the experimental and computational evidence with established literature [69, 70], we propose a plausible photocatalytic mechanism for photocatalytic H_2_O_2_ production in Phen‐TTA and O‐TTA (Figures S32 and S33). Upon visible‐light irradiation, the COFs generate e^−^–h^+^ pairs. The photogenerated electrons then migrate to the catalyst surface, where adsorbed O_2_ molecules accept an electron to form ^•^O_2_^−^. This radical subsequently reacts with protons (H^+^) in the solution to yield the OOH* intermediate, followed by another proton‐coupled electron transfer to produce H_2_O_2_. Meanwhile, a minority of O_2_ may undergo the direct 2e^−^ ORR. The enhanced π‐conjugation and rigidity of Phen‐TTA significantly accelerate charge separation, promote O_2_ adsorption/activation, and stabilize reaction intermediates, collectively contributing to its superior H_2_O_2_ generation efficiency and selectively.

The mechanism of photocatalytic methane oxidation over Phen‐TTA was subsequently investigated. Control experiments under 420 nm LED irradiation confirmed that ethanol formation was negligible in the absence of light, or in situ–generated H_2_O_2_ (Figure 4d), ruling out thermal or nonphotochemical pathways. Under the CH_4_ + H_2_O feed condition, small amount of acetic acid and ethanol were detected. This activity is likely attributed to residual O_2_ that cannot be completely eliminated from the experimental setup and/or catalyst surface, which serves as the ultimate oxygen source for oxygenate formation. Water may function as a proton‐assisted activation medium: interfacial H_2_O induces proton polarization that weakens the C─H bond of methane and optimizes CH_4_ adsorption at the catalyst surface, thereby facilitating the generation of ^•^CH_3_ radicals and their subsequent C─C coupling. In the presence of weakly reactive oxygen species (ROS) derived from trace O_2_, these intermediates are converted into ethanol and acetic acid. Compared with the CH_4_ + O_2_ system, the CH_4_ + H_2_O condition yielded a broader product distribution and a slightly higher overall activity, reflecting the role of water as a mild activation promoter that suppresses overoxidation. Nevertheless, because the reaction relies exclusively on residual trace O_2_ without an external oxidant supply, the activity remains substantially lower than that achieved in the CH_4_ + O_2_ + H_2_O system.

To further elucidate whether H_2_O_2_ is generated during photocatalytic methane oxidation over Phen‐TTA, in situ H_2_O_2_ trapping experiments were conducted under identical reaction conditions. The collected aqueous solution was semi‐quantitatively analyzed using the titanium sulfate method. As shown Figure S34, a detectable H_2_O_2_ signal was observed under the CH_4_/O_2_ atmosphere, although its intensity was relatively weaker than that obtained under a pure O_2_ atmosphere. These results indicated that H_2_O_2_ can indeed be generated concurrently with methane oxidation; however, its accumulation was suppressed in the presence of CH_4_. This behavior can be rationalized by the competitive consumption of ROS. Specifically, key intermediates involved in H_2_O_2_ formation, such as ^•^O_2_^−^, can be consumed via reactions with ^•^CH_3_ species during methane activation, thereby diverting the reaction pathway away from H_2_O_2_ accumulation. This competitive interplay is consistent with the ROS‐mediated methane oxidation pathway illustrated in Figure 4 and accounts for the diminished H_2_O_2_ signal observed experimentally.

EPR spectroscopy using DMPO as a spin trap in aqueous media detected the characteristic quartet signal of the DMPO‐^•^OH adduct for both COFs under illumination (Figure S35), verifying ^•^OH generation. To further elucidate the photodriven CH_4_‐to‐C_2_H_5_OH reaction mechanism, in situ DRIFTS was employed to track surface intermediates during methane oxidation over Phen‐TTA under 420 nm LED irradiation (Figure 4e). Adsorption bands at 883 cm^−1^ and 1307 cm^−1^ could be assigned to O_2_ and CH_4_, respectively. Upon illumination, O_2_ activation is evidenced by the emergence of ^•^O_2_^−^ (1145 cm^−1^) and ^•^OOH (1234 cm^−1^) species. With prolonged irradiation, CH_4_ activation occurs, giving rise to surface‐associated ^•^CH_3_ (1411 cm^−1^) and ^•^CH_2_ (1434 cm^−1^) intermediates, followed by the formation of oxygenated *C*_1_ species, including ^•^CH_3_OOH (1037 cm^−1^) and ^•^CH_3_O (1457 cm^−1^). Notably, the appearance of a C─O stretching vibration at 1091 cm^−1^, dominated by ^•^OCH_2_CH_3_, provides direct spectroscopic evidence for C─C coupling. The subsequent emergence of ^•^CH_2_O (1488 cm^−1^) and ^•^CH_3_CH_2_O (1376 cm^−1^) further substantiates the stepwise oxidation pathway toward selective ethanol formation. For comparison, in situ DRIFTS measurements of photocatalytic methane oxidation over O‐TTA were performed under identical conditions (Figure S36). While the types of intermediates observed are broadly consistent with those for Phen‐TTA, the corresponding signals—particularly those associated with ^•^CH_2_O, ^•^CH_3_O, ^•^CH_3_OOH, and ^•^CH_3_CH_2_O—are significantly weaker, indicating less efficient intermediate generation and transformation. This disparity likely accounts for the inferior catalytic performance of O‐TTA.

The distinct product selectivities observed for Phen‐TTA and O‐TTA may be attributed to the synergistic interplay of their intrinsic H_2_O_2_ production capabilities and topology‐induced confinement effect arising from their diverse molecular architectures. Phen‐TTA incorporates rigid phenanthroline units that construct a more rigid framework with well‐defined trirhombic pores in a *kgd‐v* topology (Scheme 1b) [16]. This structural rigidity and precise pore geometry likely create a confined microenvironment in which the nitrogen sites of the phenanthroline moieties can serve as specific adsorption and stabilization centers for ^•^CH_3_ radicals. Such spatial confinement forces radical intermediates into close proximity, thereby enhancing radical–radical coupling probability to yield *C*_2_ products such as ethanol. This behavior resembles a “microreactor” effect, whereby the framework topology directs the reaction pathway by suppressing radical diffusion, solvent quenching, and overoxidation. In contrast, O‐TTA, constructed from more flexible bipyridine linkers, forms larger and more conformationally adaptive pores in an *hcb* topology (Scheme 1b). The lack of effective spatial confinement hinders the accumulation and coupling of radical intermediates, leading instead to random collisions or overoxidation to acetic acid or CO_2_. Although both COFs can generate ^•^CH_3_ intermediates under photocatalytic conditions, topology dictates their subsequent reaction trajectories. The rigid architecture of Phen‐TTA thus acts as a structural regulator that enforces confinement and selectively promotes C─C coupling reactions.

DFT calculations based on the optimized adsorption structure of the intermediate (Figure S37) provided further insights into the photocatalytic methane oxidation process on Phen‐TTA. The C─H bond cleavage from CH_4_ to ^•^CH_3_ on Phen‐TTA exhibits an activation energy of −0.96 eV (Figure 4f). The subsequent formation of ^•^CH_3_OH through the reaction of the generated ^•^CH_3_ with ^•^OH constitutes the rate‐determining step, followed by coupling with another ^•^CH_3_ to form C_2_H_5_OH. Accordingly, a plausible reaction pathway for photocatalytic methane‐to‐ethanol conversion is proposed (Figure 4g). Photoexcited electrons first reduce surface‐adsorbed O_2_ to ^•^O_2_^−^ or ^•^OOH, which undergo proton‐coupled electron transfer to generate in situ H_2_O_2_. This H_2_O_2_ subsequently decomposes into ^•^OH radicals via photolysis. These ROSs activate CH_4_ to form ^•^CH_3_ species. Subsequent reactions with ^•^OH/^•^OOH afford ^•^CH_3_OH/^•^CH_3_OOH intermediates, which can be further oxidized and dehydrated to ^•^CH_2_O. Finally, coupling between ^•^CH_3_ and ^•^CH_2_O yields C_2_H_5_OH. The in situ generated H_2_O_2_ thus serves as a “mild yet precise” oxidative driving force, for methane conversion, which is critical for achieving efficient, green, and selective methane valorization over Phen‐TTA.

## 3 Conclusions

In this study, we report the rational design and synthesis of two N‐heterocycle‐based COFs, Phen‐TTA and O‐TTA, constructed from phenanthroline and *o*‐bipyridine donor units, respectively, paired with a TTA acceptor motif. These donor units were strategically selected to modulate molecular planarity, π‐conjugation and framework topology, thereby enabling precise regulation of the electronic band structure and enhancing photogenerated charge separation and transport. This tailored structural engineering enables both highly efficient photocatalytic H_2_O_2_ production and selective methane oxidation under ambient conditions. Comprehensive experimental and theoretical investigations demonstrate that the fused phenanthroline motif in Phen‐TTA, assembled into a rigid *kgd‐v* topology, endows the framework with a narrower optical bandgap, lowered exciton binding energy, accelerated charge‐carrier kinetics, and superior O_2_ activation capability relative to the O‐TTA, which adopts a more flexible *hcb* topology. Consequently, Phen‐TTA achieves an H_2_O_2_ production rate of 21.5 mmol h^−1^ g^−1^ with an AQY of 5.45% at 450 nm in a BA/H_2_O biphasic system, among the highest reported for metal‐free COFs. More importantly, the in situ–generated H_2_O_2_ drives selective methane oxygenation to ethanol at a rate of 30.1 µmol h^−1^ g^−1^ under 365 nm LED irradiation over Phen‐TTA, with only trace amounts of acetic acid detected, representing a rare tandem transformation accomplished without noble‐metal co‐catalysts. These performance metrics outperform those of O‐TTA as well as many previously reported COF‐based photocatalysts, highlighting the impact of topology control on photocatalytic activity. This work provides fundamental insights into the structure–property relationships governing COF‐based photocatalysts and establishes a design paradigm for the next‐generation photocatalysts aimed at sustainable solar‐driven chemical transformations.

## Author Contributions

**Lei Wang**: investigation, validation, writing – original draft, data curation, writing – review and editing. **Zhipeng Xie**: investigation, validation, data curation, writing – original draft, writing – review and editing. **Xinyi Zeng**: investigation, validation, data curation, writing – original draft. **Hetao Xu**: investigation, validation, data curation, writing – review and editing. **Mingyue Wang**: investigation, validation, data curation, writing – review and editing. **Jie Yang**: investigation, validation, data curation, writing – review and editing. **Chao Lin**: investigation, validation, data curation, writing – review and editing. **Wandong Xing**: supervision, writing – review and editing, funding acquisition. **Zhengxiao Guo**: writing – review and editing, supervision, funding acquisition. **Xiong Chen**: conceptualization, funding acquisition, supervision, writing – review and editing, project administration.

## Conflicts of Interest

The authors declare no conflict of interest.

## Supporting information




**Supporting File**: The authors have cited additional references within the Supporting Information [1–30].

## Data Availability

The data that support the findings of this study are available from the corresponding author upon reasonable request.
