# Supplementary material for: Fused Ring Engineering Induced Topology Control in Covalent Organic Frameworks: Unlocking Promoted Photocatalytic H2O2 Production and Selective Methane Oxidation
Source: Angew Chem Int Ed Engl. 2026 Apr 13;65(22):e1288153. doi: 10.1002/anie.1288153 (PMC13206243; doi:10.1002/anie.1288153)
Supplement: Supplementary file 1 — Supporting File: The authors have cited additional references within the Supporting Information [1–30]. [file ANIE-65-e1288153-s001.docx]

**Supporting Information**

**Fused Ring Engineering Induced Topology Control in Covalent Organic Frameworks: Unlocking Promoted Photocatalytic H_2_O_2_ Production and Selective Methane Oxidation**

Lei Wang,^[a][b][+]^ Zhipeng Xie,^[c][+]^ Xinyi Zeng,^[a][b][+]^ Hetao Xu,^[a][b][+]^ Mingyue Wang,^[a][b]^ Jie Yang,^[c]^ Chao Lin,^[a][b]^ Wandong Xing,*^[a][b]^ Zhengxiao Guo,*^[c]^ and Xiong Chen*^[a][b]^

[a] L. Wang, X. Zeng, Dr. H. Xu, M. Wang, C. Lin, Prof. W. Xing, Prof. X. Chen

State Key Laboratory of Chemistry for NBC Hazards Protection

College of Chemistry, Fuzhou University

Fuzhou 350116, P. R. China

E-mail: xwd@fzu.edu.cn; chenxiong987@fzu.edu.cn

[b] L. Wang, X. Zeng, Dr. H. Xu, M. Wang, C. Lin, Prof. W. Xing, Prof. X. Chen

State Key Laboratory of Photocatalysis on Energy and Environment, and Key Laboratory of Advanced Carbon-Based Functional Materials

College of Chemistry, Fuzhou University

Fuzhou 350116 (P. R. China)

[c] Dr. Z. Xie, J. Yang, Prof. Z. Guo

Department of Chemistry, The University of Hong Kong

Hong Kong SAR 99907, P. R. China

E-mail: zxguo@hku.hk

[+] These authors contributed equally to this work.

**Contents**

**Section 1. Materials and Methods.**

**Section 2. Synthetic Procedures.**

**Section 3. Supporting Figures.**

**Section 4. Supporting Tables.**

**Section 5. References.**

Section 1. Materials and Methods.

Materials.

The 4,4′-(1,10-Phenanthroline-2,9-diyl)bis[benzaldehyde], (2,2'-Bipyridine)-6,6'-dicarbaldehyde, 4,4',4''-(1,3,5-Triazine-2,4,6-triyl)trianiline, 2,9-Dibromo-1,10-phenanthroline, tetrakis(triphenylphosphine)palladium(0) and 4-(4,4,5,5-Tetramethyl-1,3,2-dioxaborolan-2-yl)benzaldehyde were purchased from Shanghai Bide Pharmatech Co., Ltd. Potassium carbonate, 1,4-dioxiane, mesitylene (M), EtOH and acetic acid (AcOH) were purchased from Shanghai Macklin Biochemical Co., Ltd. Dichloromethane (DCM), acetone and tetrahydrofuran were purchased from Sinopharm Chemical Reagent Ltd. All chemical reagents were used purchased without further purification.

Characterizations.

The X-ray diffraction (XRD) pattern of samples was recorded on a Rigaku MiniFlex 600 machine with Cu Kαradiation (λ = 1.54056 Å) at room temperature. Fourier Transform Infrared Spectroscopy (FT-IR) were recorded on a Fourier transform infrared (FTIR, Thermo Nicolet Magna 670) spectroscope. ^1^H nuclear magnetic resonance (NMR) spectra were analyzed on a JNM3ECZ600R 600 MHz NMR instrument. The solid-state ^13^C CP/MAS NMR experiments were performed on a Bruker Advance III 500 spectrometer. X-ray photoelectron spectroscopy (XPS) measurements were obtained on Thermo ESCALAB250 instrument with a monochromatized Al Kα line source (200 W). Nitrogen sorption isotherms were measured at 77 K on a Micromeritics ASAP 2460 analyzer. Transmission electron microscope (TEM) operated at a FEI Talos F200S G2 at 200 kV. The UV-Vis diffuse reflectance spectra (UV-Vis DRS) were collected on a UV-2600 UV-vis spectrophotometer (Shimadzu) with a wave range of 200-800 nm. Steady-state photoluminescence (PL) emission spectra and PL decay spectra at room temperature were recorded using HORIBA Instruments FLS1000 spectrophotometer. Temperature-dependent PL spectra from 160 K to 300 K were recorded on an Edinburgh FI/FSTCSPC 920 spectrophotometer. Electron paramagnetic resonance (EPR) measurements were performed on a Bruker model A 300 spectrometer.

**Photocatalytic H_2_O_2_ production.**

In pure water system: The COF (10 mg) was added to a pure water (20 mL) reaction system in a glass bottle (50 mL), and then ultrasonicated for 10 min to disperse the COF. The bottle opening was then sealed with a two-way valve connected to an oxygen balloon, creating an oxygen atmosphere in the reaction system. During the reaction, the temperature of the reaction solution was maintained at room temperature by circulating cooling water, and the solution was irradiated with 420 nm LED monochromatic light.

In water/BA biphasic system: The COF (10 mg) was added to a two-phase reaction system composed of water (18 mL) and benzyl alcohol (2 mL) in a glass bottle (50 mL), and all other experimental operations were consistent with those under pure water conditions.

**H_2_O_2_ detection methods.**

The H_2_O_2_ amount was determined using a Shimadzu UV-1780 UV-Vis spectrophotometer by measuring its absorbance on the basis of the formation of the yellow complex Ti (IV)-H_2_O_2_. The Ti^4+^ abundant solution will become a yellow complex when it interacts with H_2_O_2_ and UV−Vis measurement was done at 410 nm to colorimetrically determine the concentration of H_2_O_2_ as shown in Equation S1.

Ti (SO_4_)_2_ + H_2_O_2_ → H_2_[Ti_2_(SO_4_)_2_] Equation S1

The specific procedure was as follows: the reaction solution was filtered, 5 mL were transferred to a volumetric flask and 2 mL of Ti(SO_4_)_2_ solution were added. Deionised water was then added until the final volume of the mixture reached 25 mL. The volumetrically adjusted mixture was then transferred to a cuvette for measurement.

**Calibrations for the measurements of the H_2_O_2_ solution.**

H_2_O_2_ solutions with the concentrations of 0.4, 0.8, 1.2, 1.6, 2 mmol/L were prepared. The Ti(SO_4_)_2_ solution was used as the color indicator before setting volume. The mixture solution was shaken and then kept still for 2 minutes. UV-vis spectra of the solution were recorded by using a UV-vis spectrophotometer at 410 nm. The linear relationship between H_2_O_2_ concentration and the absorption intensity was established as follows.

**
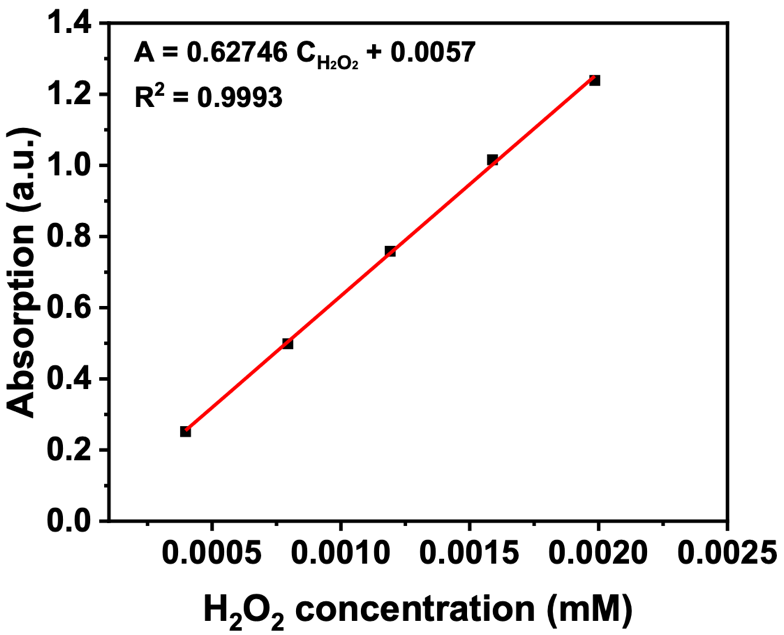
**

**Photocatalytic methane oxidation.**

Photocatalytic methane oxidation reactions were performed in a custom flat-plate batch reactor with an integrated quartz window, constructed from stainless steel and ceramic to reduce reactant adsorption and ensure structural stability (Figure S21). A 365 nm/420 nm LED light source was used, with its emitting surface fixed 5 cm vertically from the quartz window for uniform catalyst illumination. 5 mg of as-prepared catalyst was vacuum-deposited onto a glass fiber membrane, which was placed in the 18 mL chamber to enhance reactant contact. Rubber gaskets and mechanical clamping ensured leak-tight sealing, while stainless steel tubing prevented gas contamination. Pre-humidified (25 ℃ deionized water) 20% CH_4_/Ar and 20% O_2_/N_2_ (BOC-grade gases) were fed at a total 40 sccm flow rate via a Bronkhorst mass flow controller. Gaseous products were monitored online by GC; liquid products were condensed in a cold trap and analyzed via solution NMR.

**Photoelectrochemical measurements.**

Preparation of catalyst electrodes: Take 2.5 mg of catalyst sample and ultrasonically disperse it in 450 μL of DMF solution. Add 50 μL of Nafion dispersion solution as a binder. Dispense 20 μL of the dispersion solution in multiple droplets onto the conductive surface of fluorine-doped tin oxide (FTO) conductive glass (2 × 1 cm^2^).

Photoelectrochemical testing: Evaluate the photoelectrochemical properties of the sample using a standard three-electrode cell system. This includes a catalyst electrode, a reference electrode, and a counter electrode. The reference electrode is an Ag/AgCl electrode in 3 M KCl solution, the counter electrode is a Pt sheet, the electrolyte is 0.2 M Na_2_SO_4_ solution (pH = 6.8), and the light source is a Xenon lamp.

**Rotating disk electrode (RDE) measurements.**

To prepare the working electrode for RDE measurements, 2 mg COF samples were dispersed in 0.7 mL of EtOH containing 0.1mL of Nafion by 5 min ultrasonication. Then, 10 μL of the slurry was dripped onto a glassy carbon rotating disk electrode and dried at room temperature. In this measurement, the as-prepared RDE, platinum plate, and Ag/AgCl were used as working electrode, counter electrode, and reference electrode, respectively, with an O_2_-saturated 0.1 M KOH aqueous solution as the electrolyte. The linear sweep voltammograms (LSV) were obtained under room temperature with a scan rate of 5 mV s^-1^ and different rotating speeds ranging from 400 rpm to 1600 rpm. The average electron transfer numbers (n) were calculated by the Koutecky-Levich equation:


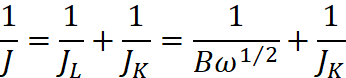


B=0.62nFC_0_D_0_^2/3^V^-1/6^

Where J is the measured current density, J_K_ refers to the kinetic current density, J_L_ is the limiting diffusion current density, n is the transfer electron number, F represents the Faraday constant (96485 C mol^-1^), and C_0_ is saturated oxygen concentration in electrolyte (1.26 × 10^-3^ M). D_0_ is the diffusion coefficient of O_2_ (1.93 ×10^-5^ cm^2^ s^-1^), V is the kinetic viscosity of the electrolyte (0.0109 cm^2^ s^-1^), and ω is the rotation rate (rad s^-1^) of the electrode.

***In-situ* DRIFTS measurements.**

*In-situ* DRIFTS measurements were performed on a Bruker VERTEX 80v instrument. Before the measurement, the samples were degassed at 363 K for 60 minutes. The baseline was obtained after continuous steam-saturated O_2_ flowing for 60 minutes in the dark. Light source was from a FX300 (Beijing Perfect Light Technology Co., Ltd., Beijing, China).

**AQY measurement for H_2_O_2_ production**

The apparent quantum yield (AQY) for H_2_O_2_ evolution was measured under the illumination of monochromatic LED lamps with band pass filter of 420, 450,520 and 600 nm for 1 hour. The lamp spacing is 5 cm, the light intensities were 35.3, 42.4, 19.2 and 17.3 mW cm^-2^, respectively. The irradiation area was controlled as 4 cm^2^. The AQY values were calculated using the following equation:


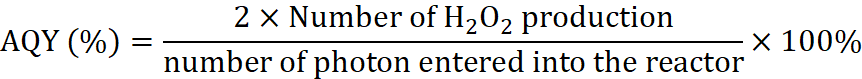


The number of photon entered into the reactor is:


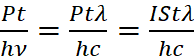

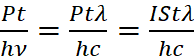


Where , *N*_A_ is Avogadro constant (6.022 ×10^23^ mol^-1^), *h* is the Planck constant (6.626 × 10^-34^ J·s), *c* is the speed of light (3.0 × 10^8^ m s^-1^), *S* is the irradiation area (cm^2^), *I* is the intensity of irradiation light (W cm^-2^), *t* is the photoreaction time (s), and *λ* is the wavelength of the monochromatic light (m).

DFT calculations

The calculations are performed based on spin-polarized density functional theory (DFT) implemented in Vienna Ab initio Simulation package (VASP) with the Projector Augmented Wave (PAW) method.^[1]^ The exchange-correlation term was described using the Perdew-Burke-Ernzerhof (PBE) functional. DFT-D3 correction method was used to describe the van der Waals interaction.^[2]^ The kinetic cutoff energy of the plane wave expansion was set to 450 eV. Convergence criteria of energy and force for all geometry structures were fixed to 10^-4^ eV and 0.05 eV Å^-1^.

The monolayer models of two COFs were used for all the calculations. The Brillouin zone was sampled using a gamma (1 × 1 × 1) point. The Gibbs free energy was calculated with reference to the computational hydrogen electrode (CHE) proposed by Nørskov et al.^[3]^ The change of Gibbs free energy (ΔG) was computed as follows:

ΔG = ΔE + ΔE_ZPE_ − TΔS

where ΔE, ΔE_ZPE_, and ΔS represent the difference value of total energy, zero potential energy, and entropy, respectively, and T is 298.15 K. ΔE_ZPE_ and ΔS can be obtained according to the calculated vibrational frequencies. The VASPKIT program was used to keep the thermal correction to Gibbs free energy for all adsorbates.^[4]^

**Section 2. Synthetic Procedures.**

**COFs synthesis.**

**O**-**TTA:** A mixture of TTA (92 mg, 0.26 mmol) and O-BPY (82.7 mg, 0.39 mmol) was suspended in mesitylene/dioxane (5.1 mL/0.9 mL) with acetic acid as catalyst (6 M, 0.2 mL) in a 10 ml Schlenk tube. After sonication for 5 min, the mixture was degassed via freeze-pump-thaw for three cycles at 77 K in a liquid N_2_ bath and further heated at 120 °C for 3 days. When cooled at room temperature, the precipitate was collected by filtration and washed by THF, and acetone three times, respectively. The solids obtained were purified by Soxhlet extraction with tetrahydrofuran. The final product was dried overnight in a vacuum oven at 60 °C to yield light yellow powder O-TTA.

**Phen-TTA:** A mixture of TTA (14.18 mg, 0.04 mmol) and Phen (23.31 mg, 0.06 mmol) was suspended in mesitylene/EtOH (0.5 mL/1.5 mL) with acetic acid as catalyst (6 M, 0.2 mL) in a 10 ml Schlenk tube. After sonication for 5 min, the mixture was degassed via freeze-pump-thaw for three cycles at 77 K in a liquid N_2_ bath and further heated at 120 °C for 3 days. When cooled at room temperature, the precipitate was collected by filtration and washed by THF, and acetone three times, respectively. The solids obtained were purified by Soxhlet extraction with tetrahydrofuran. The final product was dried overnight in a vacuum oven at 60 °C to yield yellow powder Phen-TTA.

**Section 3. Supporting Figures.**


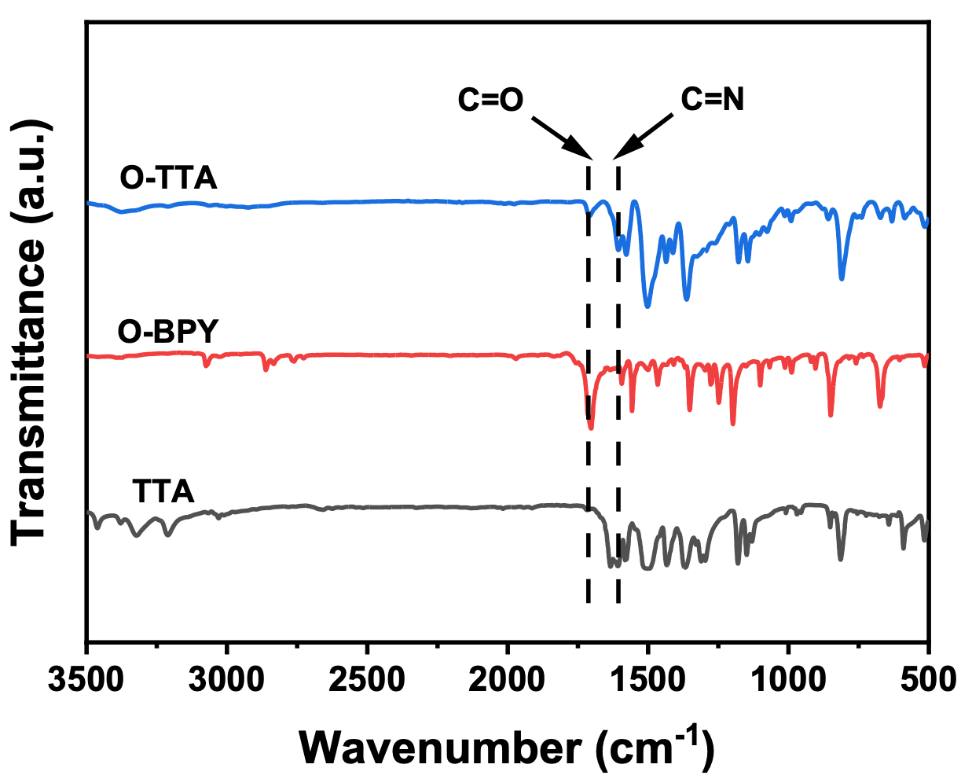


**Figure S1.** FT-IR spectra comparison of O-TTA (blue), O-BPY (red) and TTA (black).


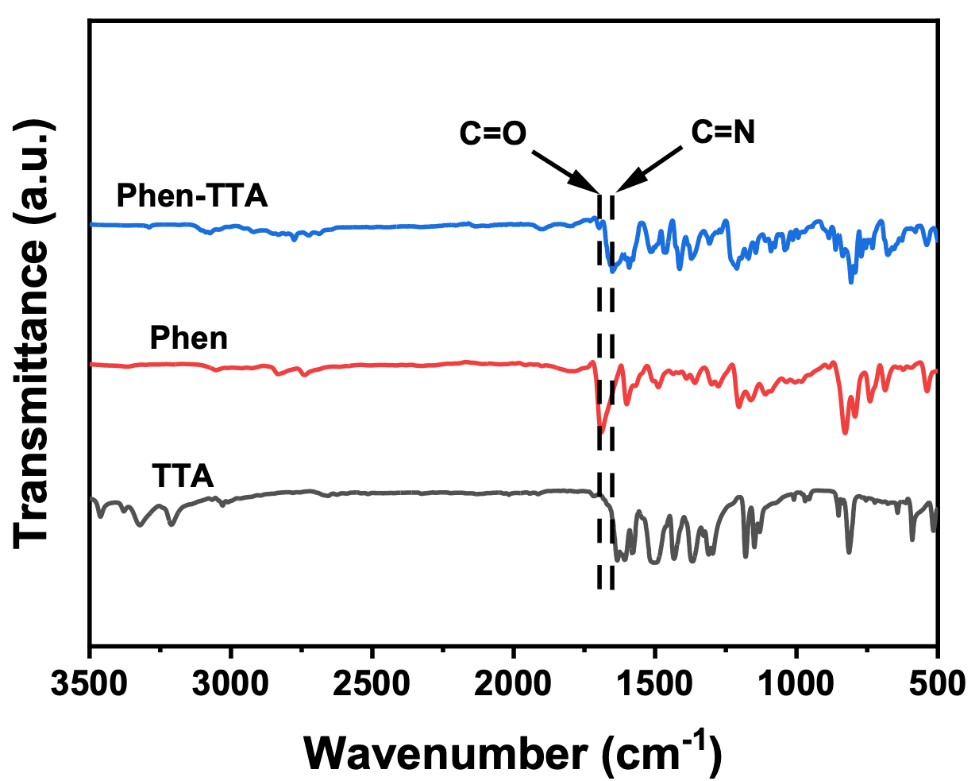


**Figure S2.** FT-IR spectra comparison of Phen-TTA (blue), Phen (red) and TTA (black).


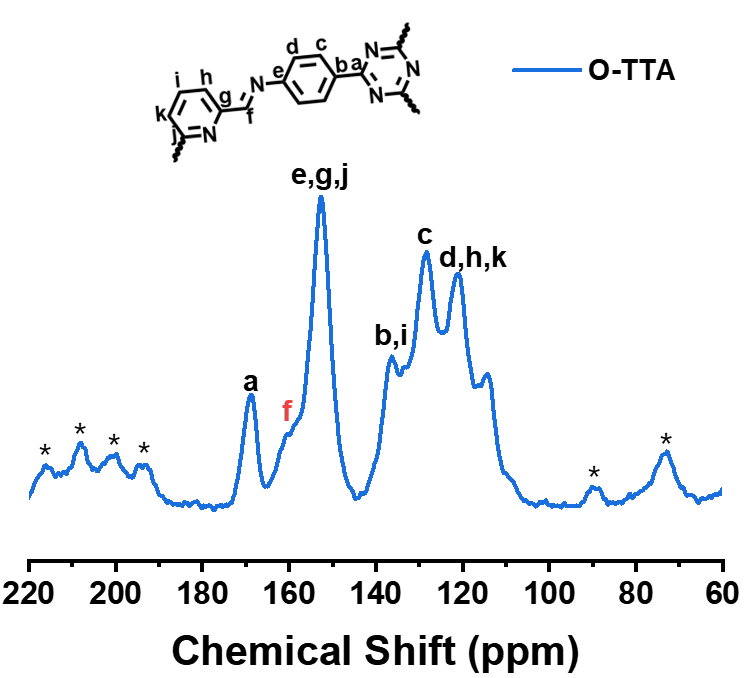


**Figure S3.** Solid state ^13^C CP/MAS NMR spectra of O-TTA (“*” denotes the spinning sidebands).


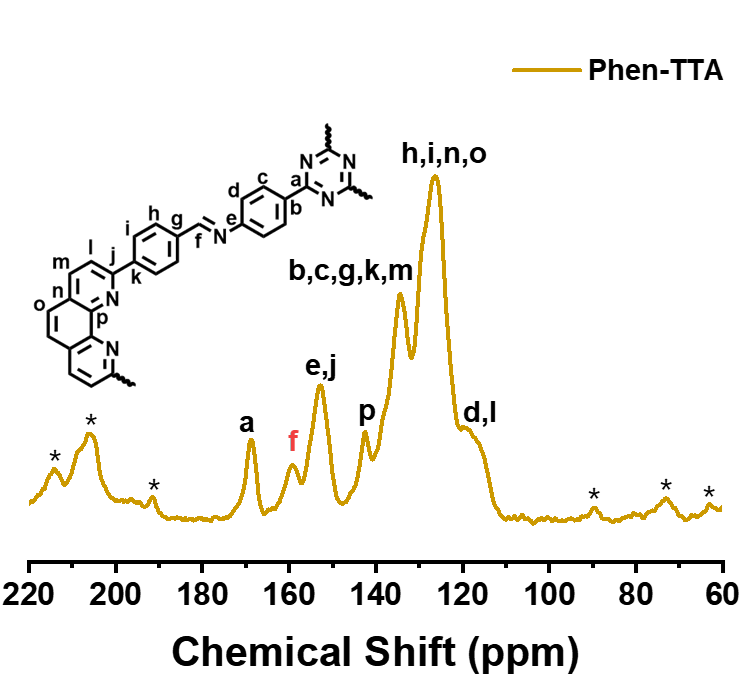


**Figure S4.** Solid state ^13^C CP/MAS NMR spectra of Phen-TTA (“*” denotes the spinning sidebands).


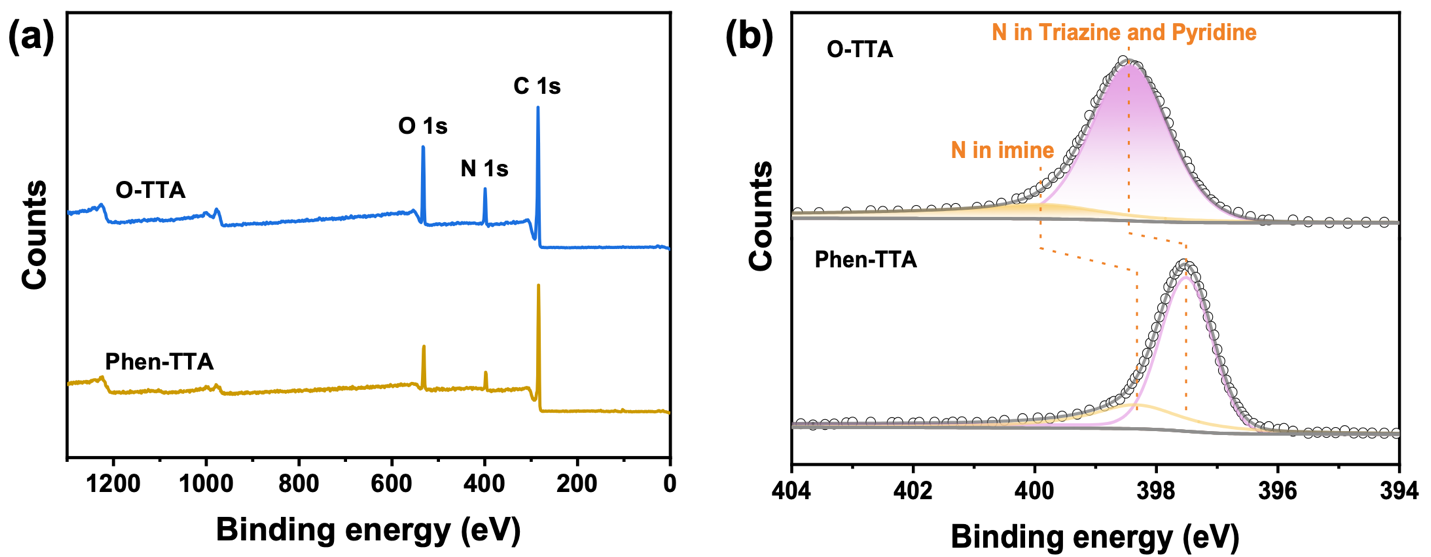


**Figure S5**. XPS spectra: (a) survey XPS spectra and (b) N 1s XPS spectra of O-TTA and Phen-TTA.


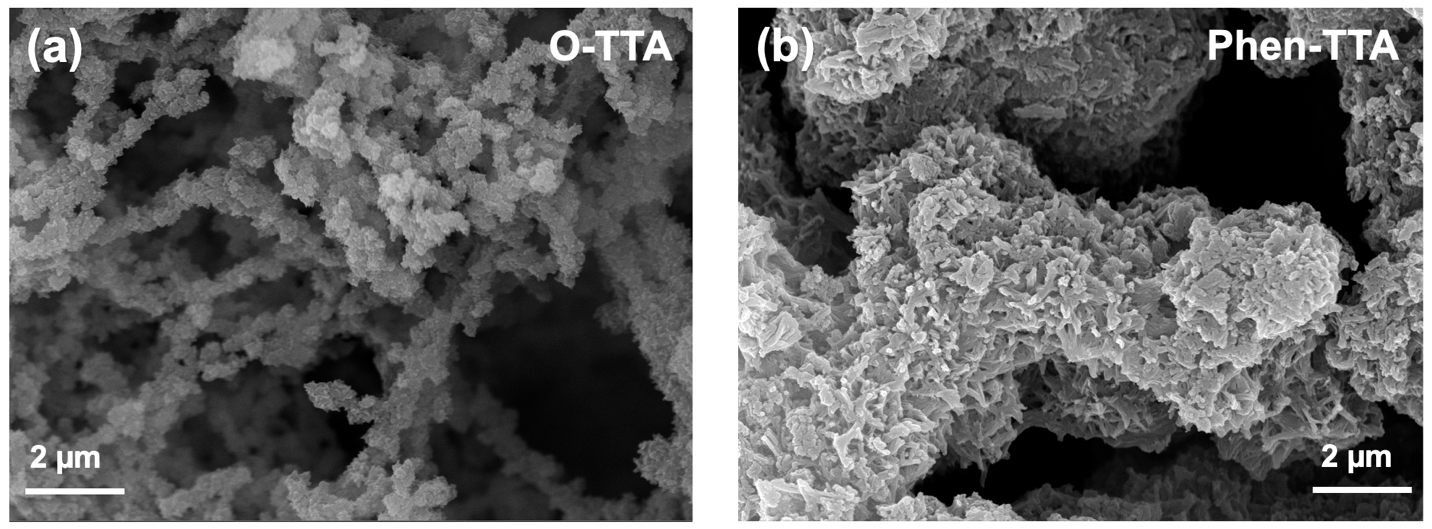


**Figure S6**. SEM images of (a) O-TTA and (b) Phen-TTA.


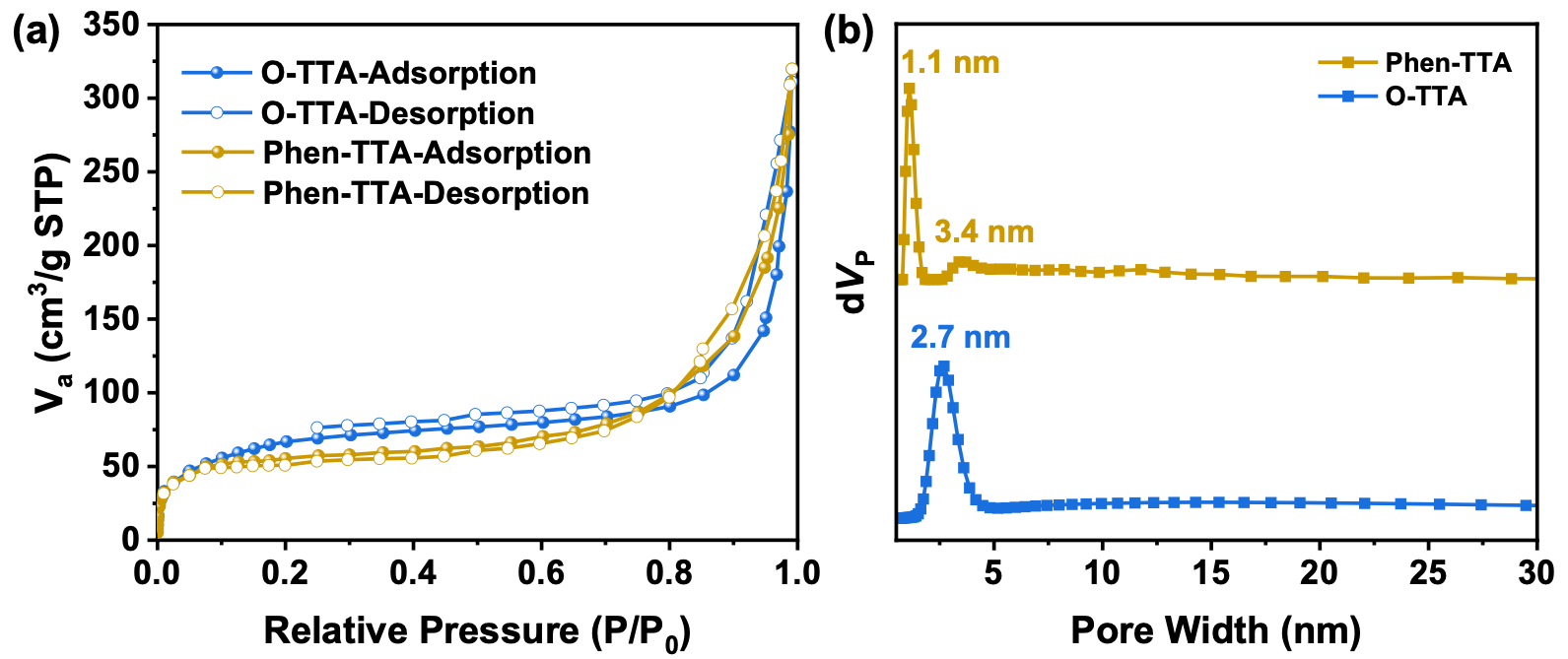


**Figure S7.** (a) N_2_ sorption isotherms and (b) pore size distribution of O-TTA and Phen-TTA at 77 K.


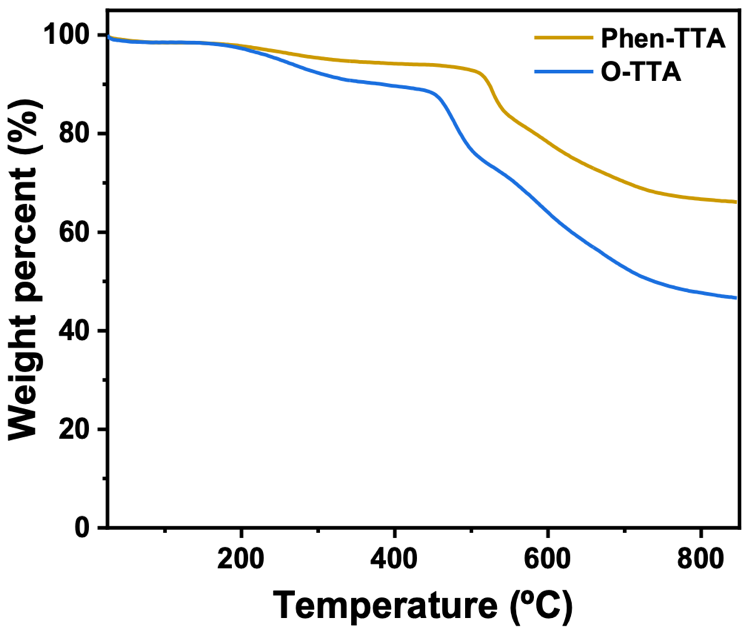


**Figure S8.** Thermogravimetric analysis of Phen-TTA and O-TTA in N_2_ atmosphere.


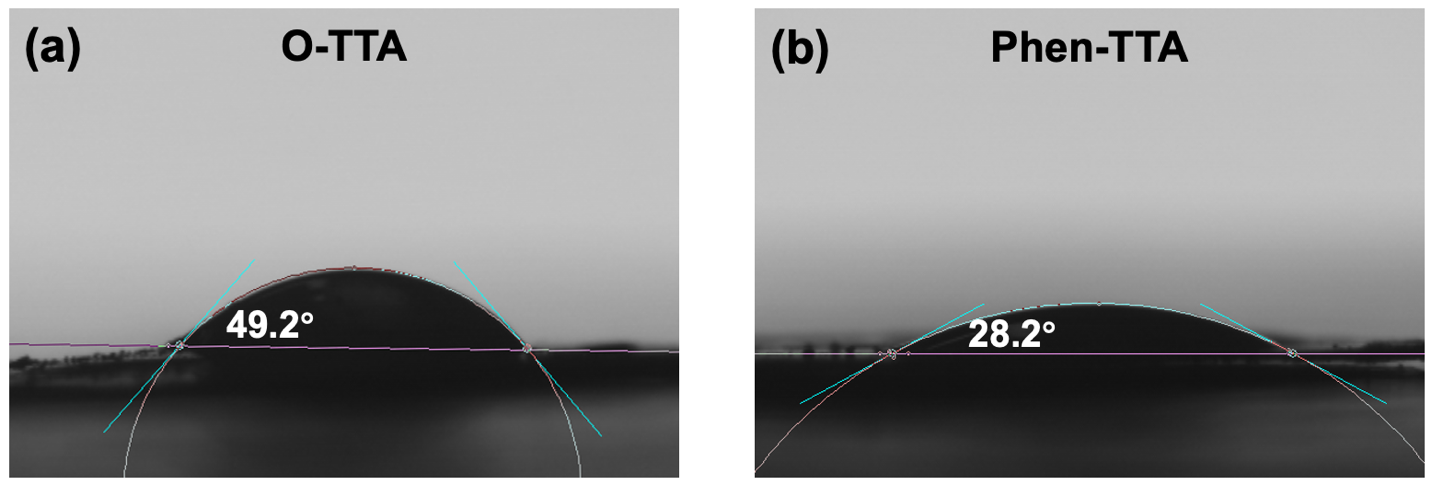


**Figure S9.** Water contact angles of (a) O-TTA and (b) Phen-TTA.


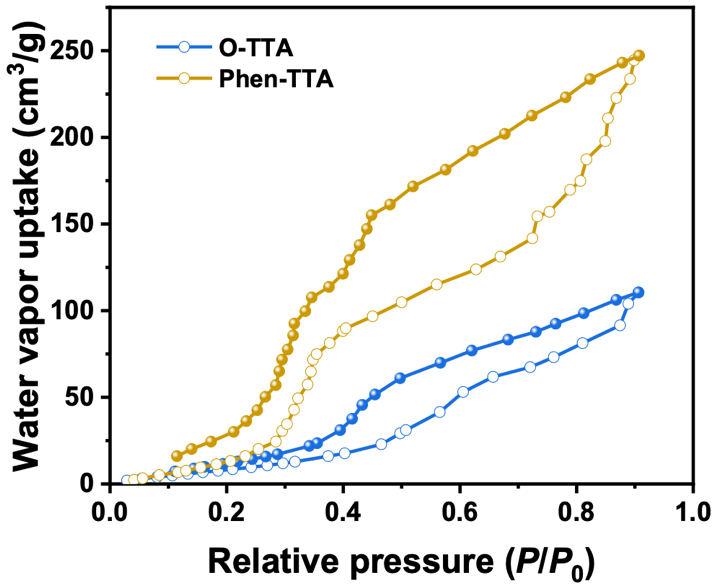


**Figure S10.** Water vapor adsorption profiles of O-TTA and Phen-TTA.


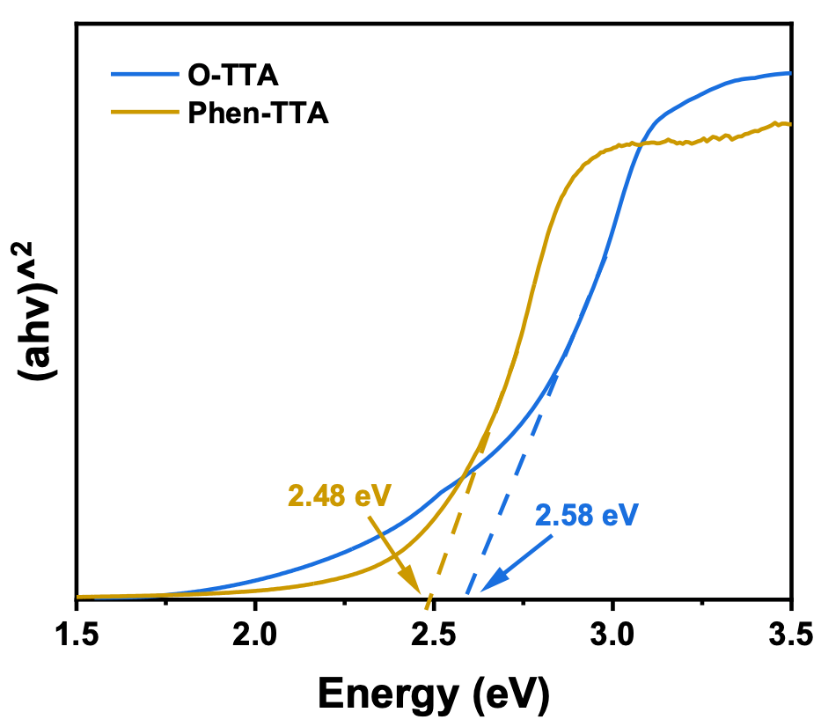


**Figure S11.** Kubelka-Munk-transformed reflectance spectra of O-TTA and Phen-TTA.


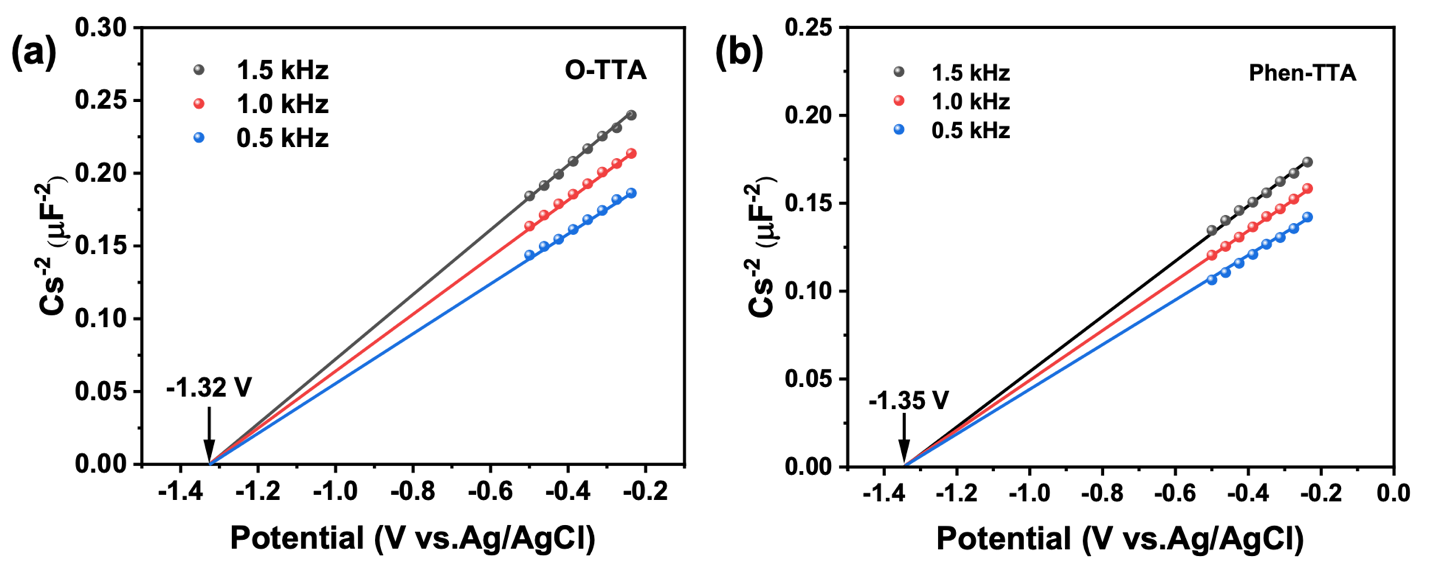


**Figure S12**. Mott-Schottky curves of (a) O-TTA and (b) Phen-TTA.


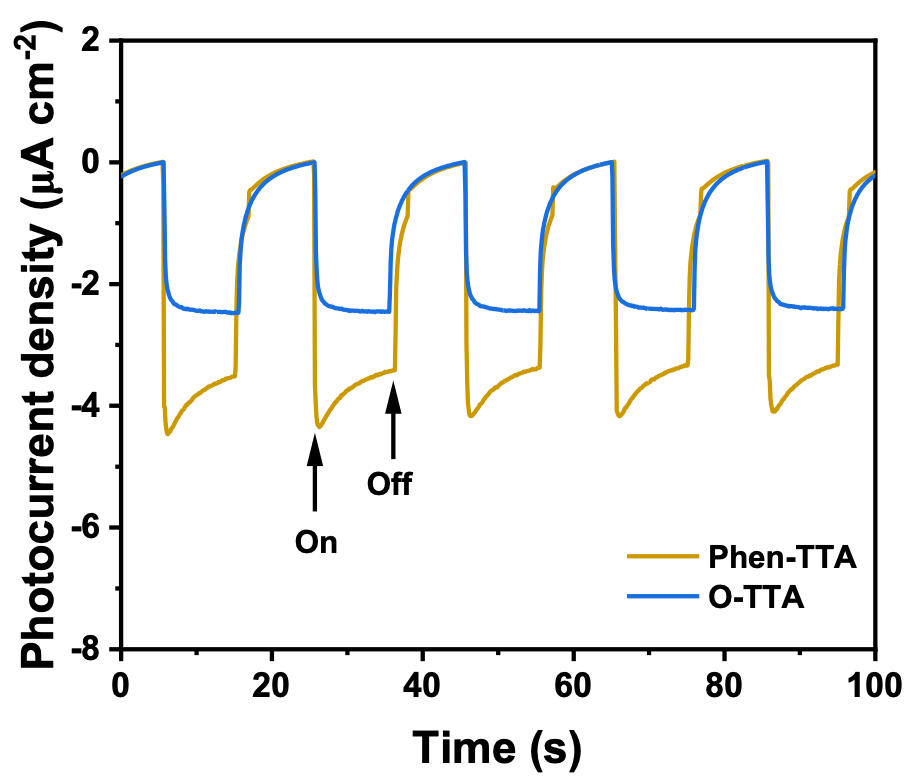


**Figure S13.** Transient photocurrent responses of O-TTA and Phen-TTA.


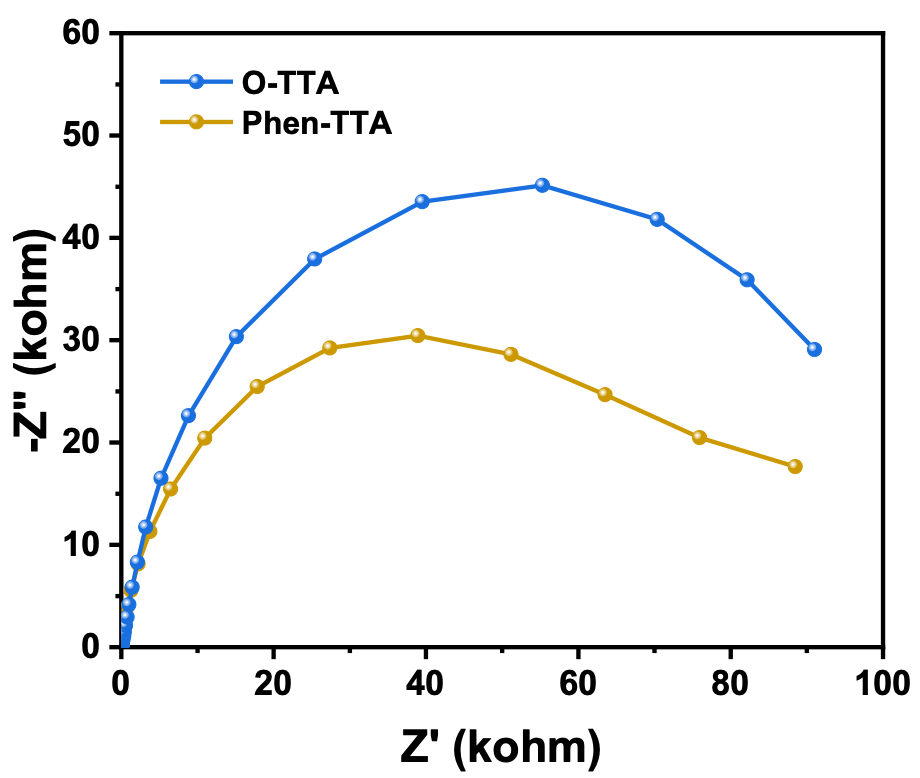


**Figure S14.** Electrochemical impedance spectroscopy (EIS) Nyquist plots of O-TTA and Phen-TTA.


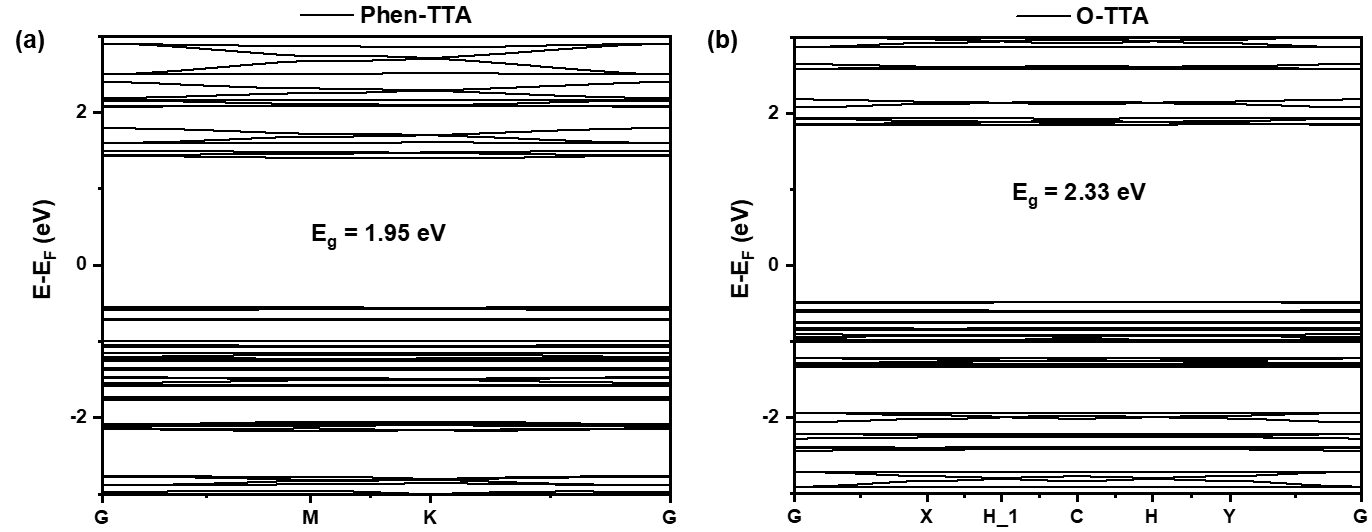


**Figure S15.** The highest occupied molecular orbital-lowest unoccupied molecular orbital (HOMO-LUMO) energy gap values calculated for the two structures via density functional theory (DFT).


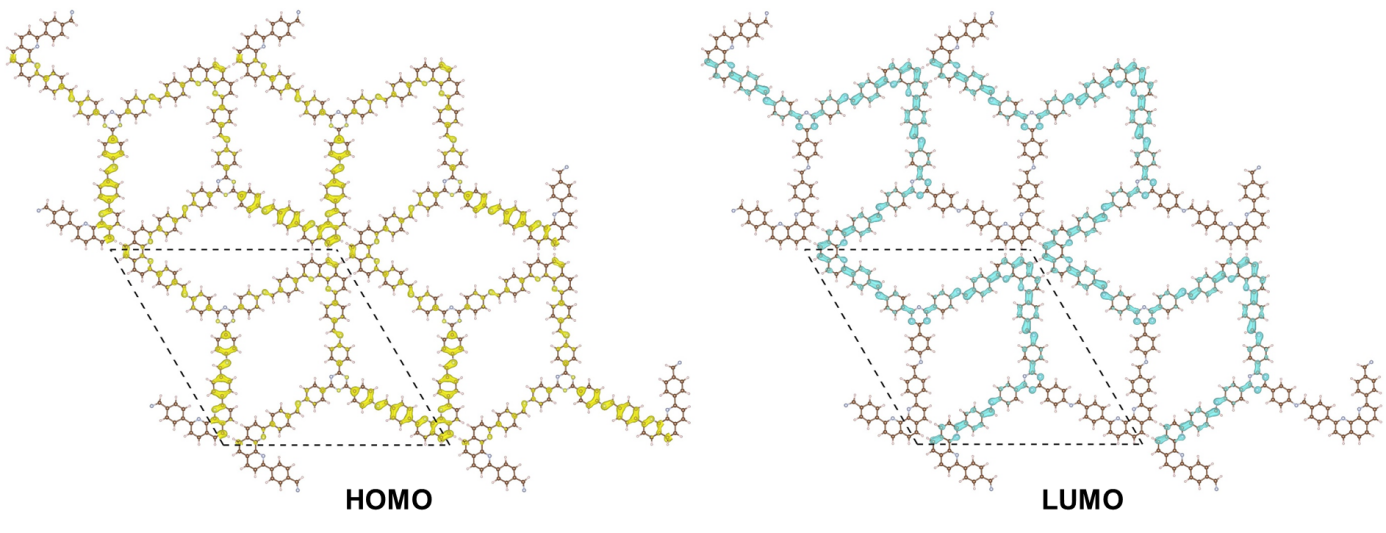


**Figure S16.** Theoretical HOMO/LUMO of the simulated fragmental structures of Phen-TTA.


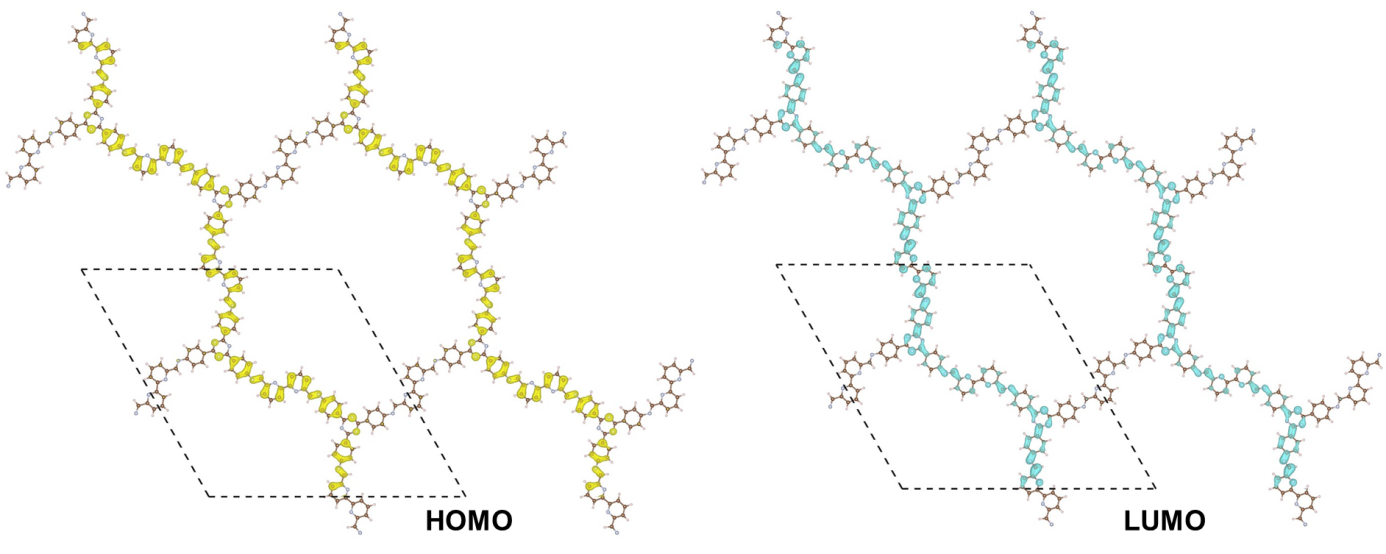


**Figure S17.** Theoretical HOMO/LUMO of the simulated fragmental structures of O-TTA.

**
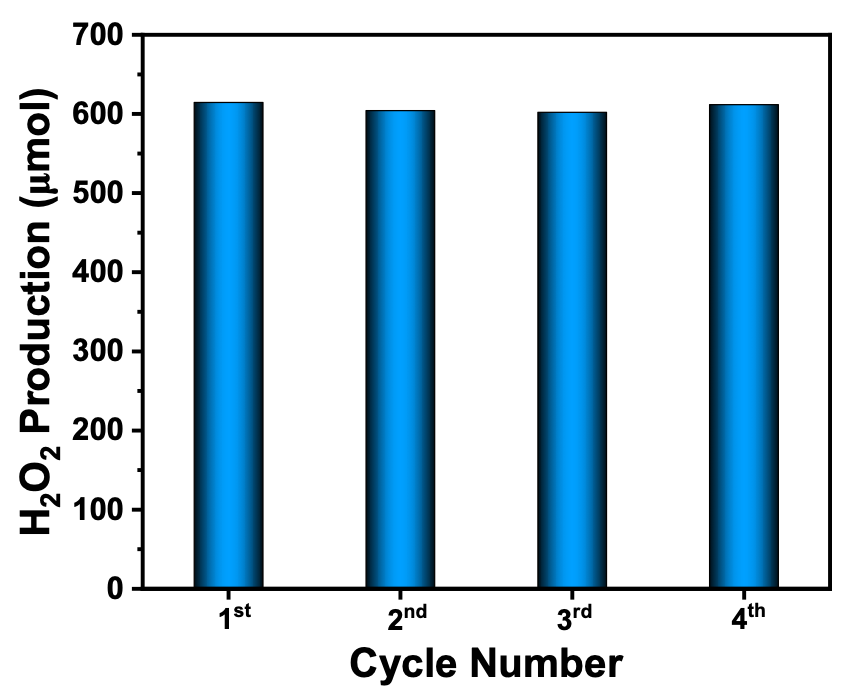
**

**Figure S18.** Cyclic stability experiment of BA as sacrifice agent over 2 h.

**
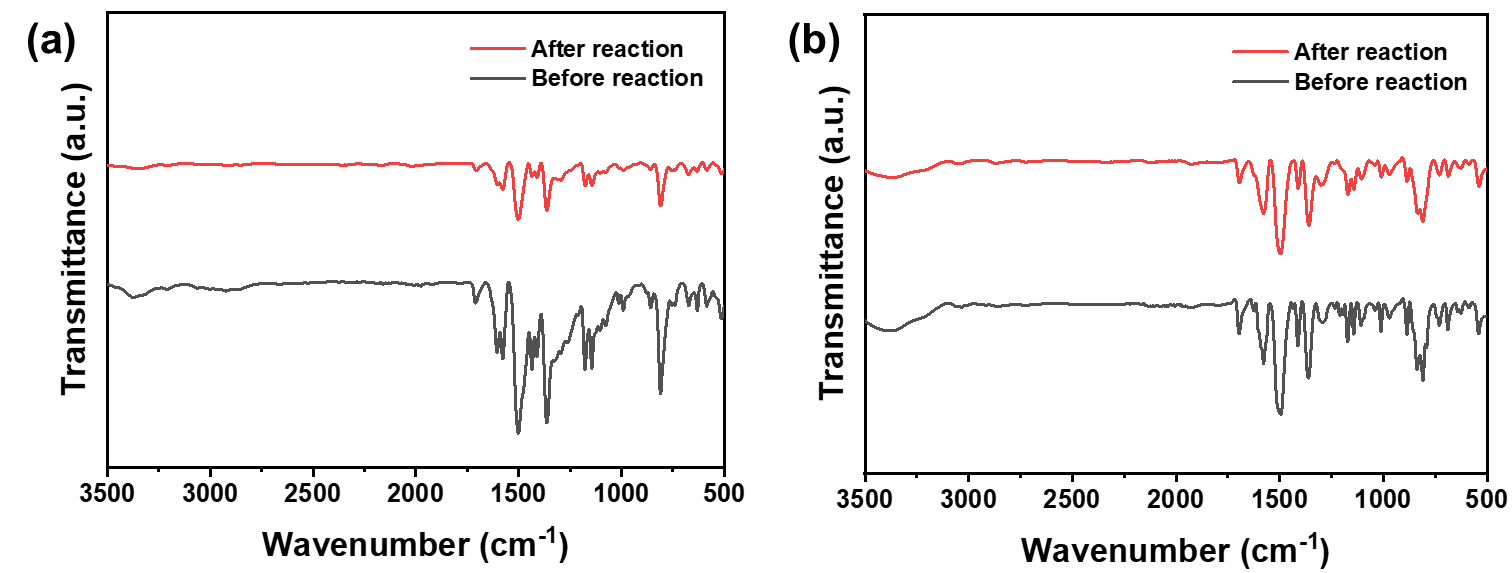
Figure S19.** FT-IR spectra of (a) O-TTA and (b) Phen-TTA before and after reaction.


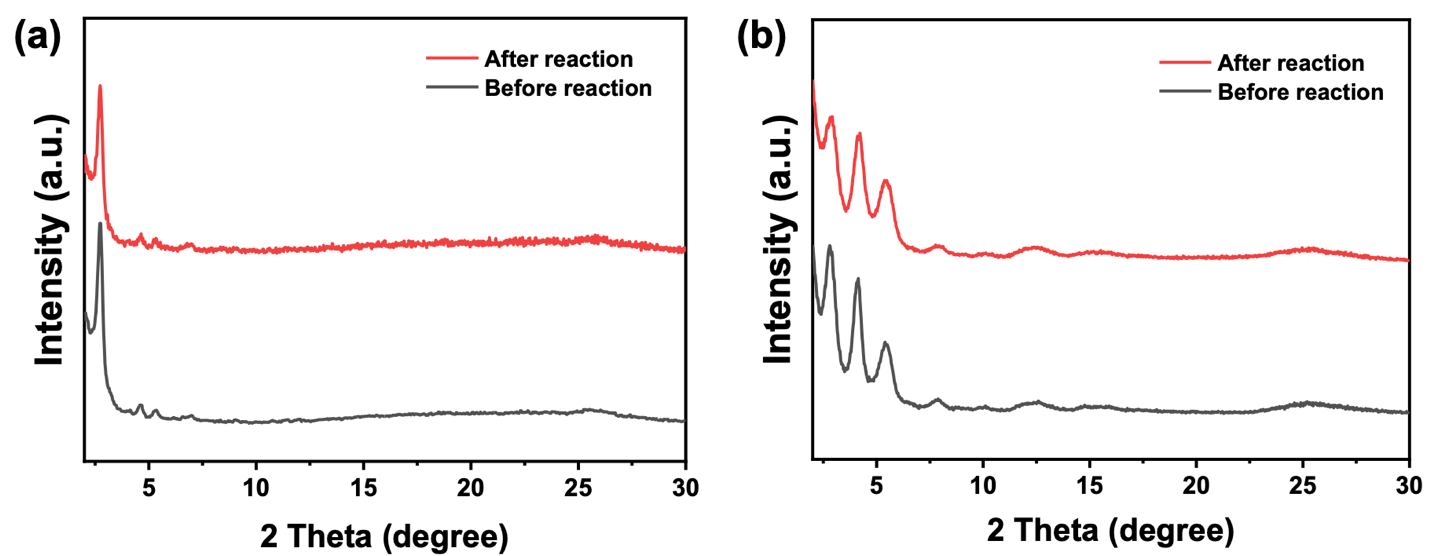


**Figure S20.** PXRD patterns of (a) O-TTA and (b) Phen-TTA before and after reaction.

**
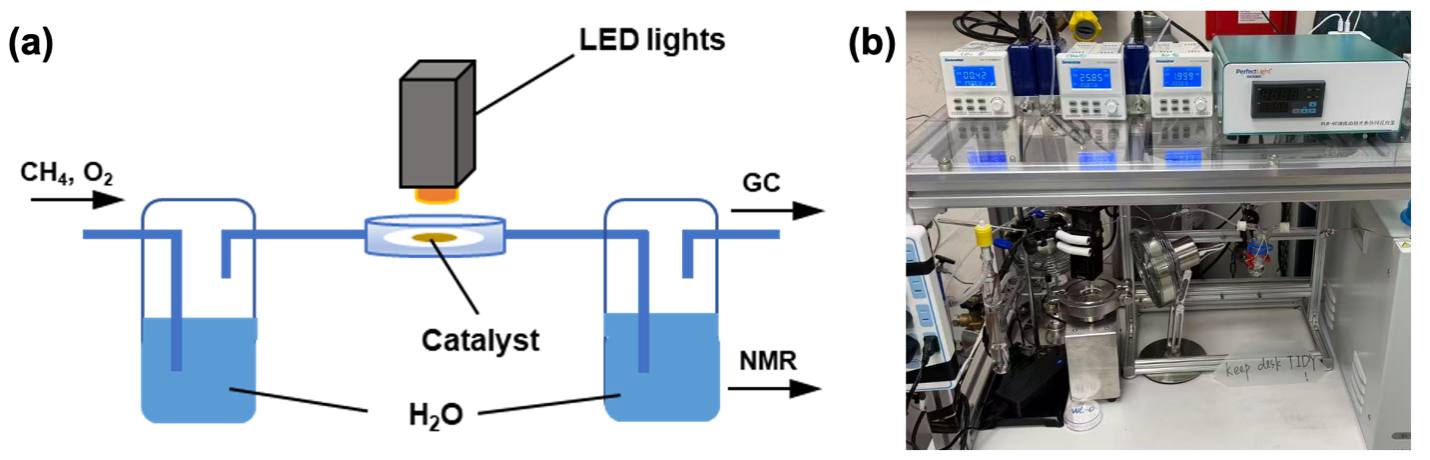
**

**Figure S21.** (a) Schematic illustration and (b) digital image of the reaction setup.

**
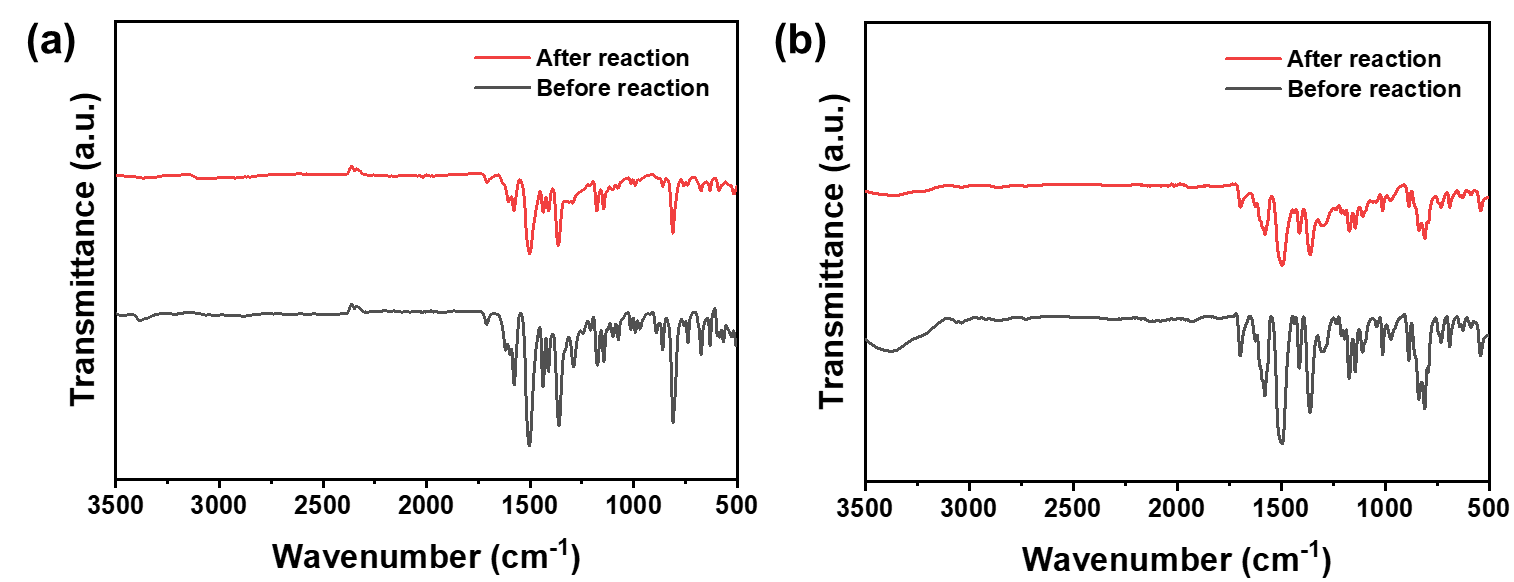
**

**Figure S22.** FT-IR spectra of (a) O-TTA and (b) Phen-TTA before and after methane transformation.

**
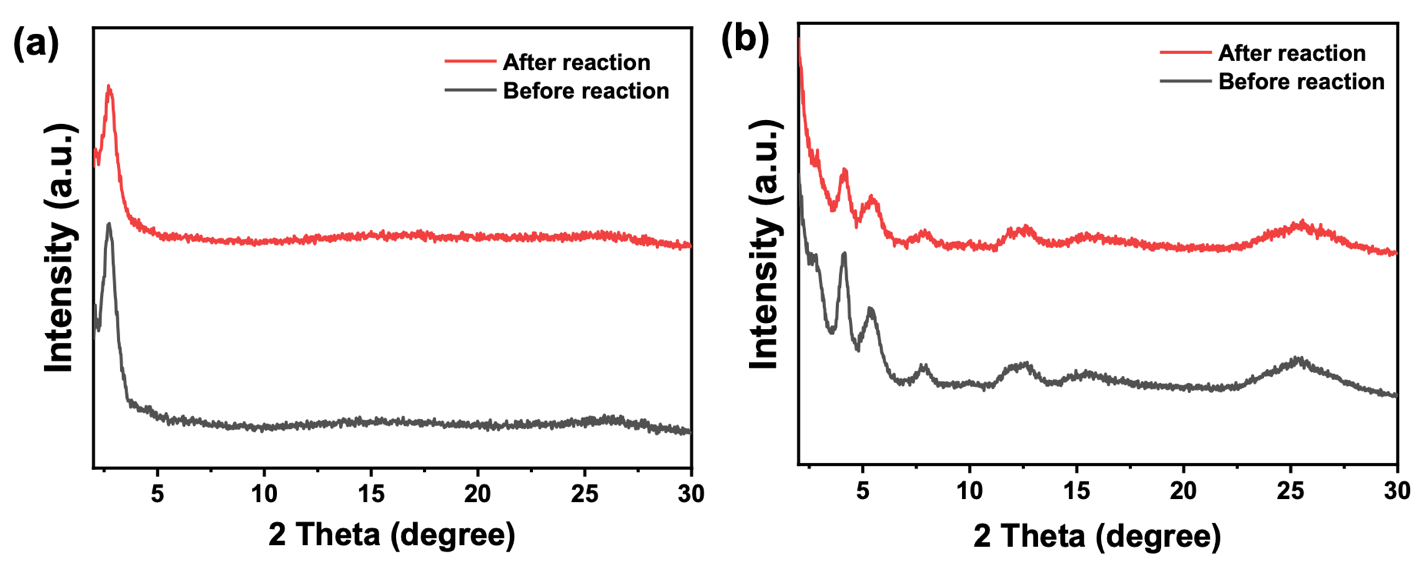
**

**Figure S23.** PXRD patterns of (a) O-TTA and (b) Phen-TTA before and after methane transformation.


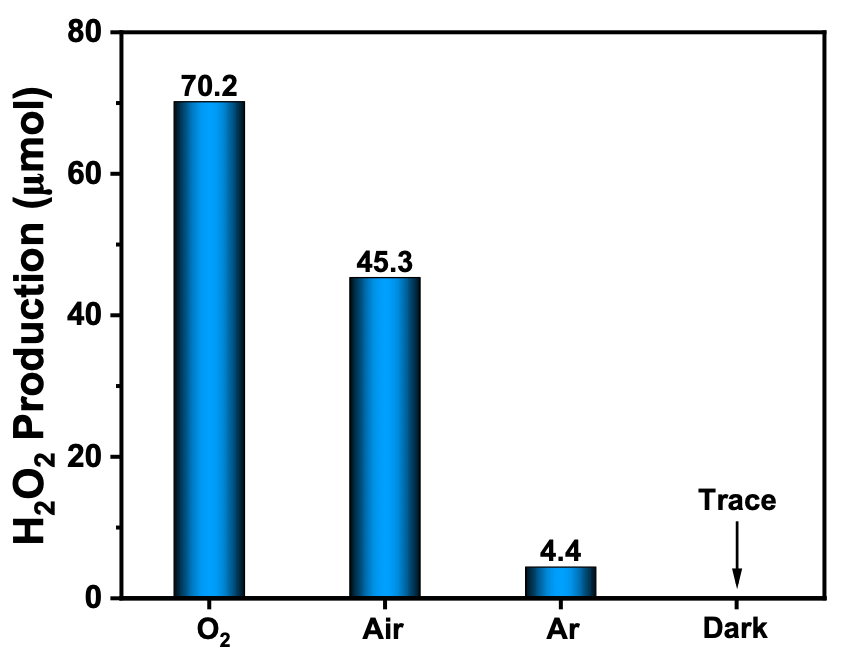


**Figure S24.** Photocatalytic H_2_O_2_ production activity of Phen-TTA under different conditions over 0.5 h.


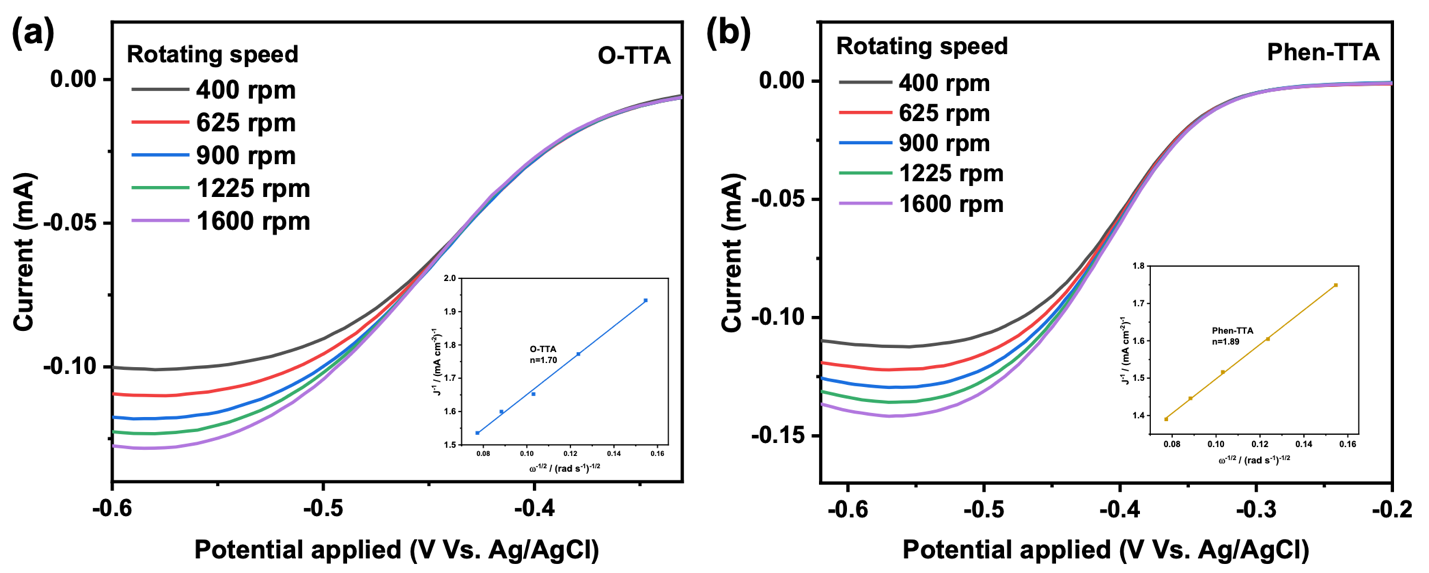


**Figure S25.** LSV curves for (a) O-TTA and (b) Phen-TTA detected by RDE. The inset shows the fitted K-L curve plot.

**
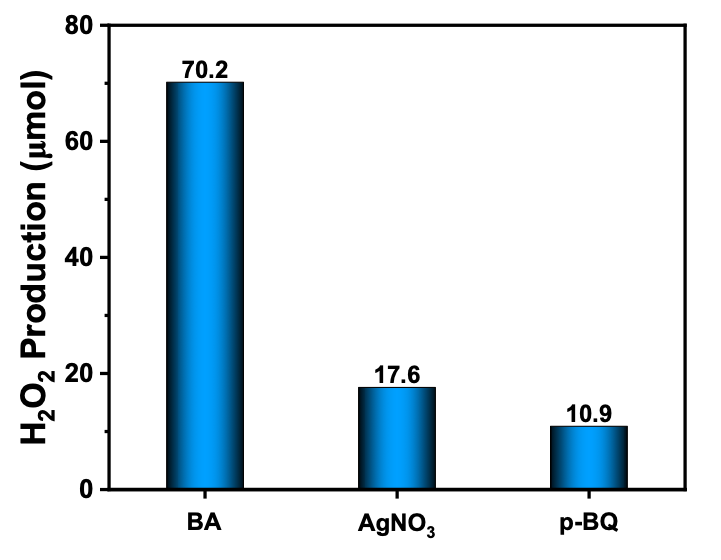
**

**Figure S26.** Photocatalytic H_2_O_2_ production activity using Phen-TTA with different scavengers over 0.5 h.


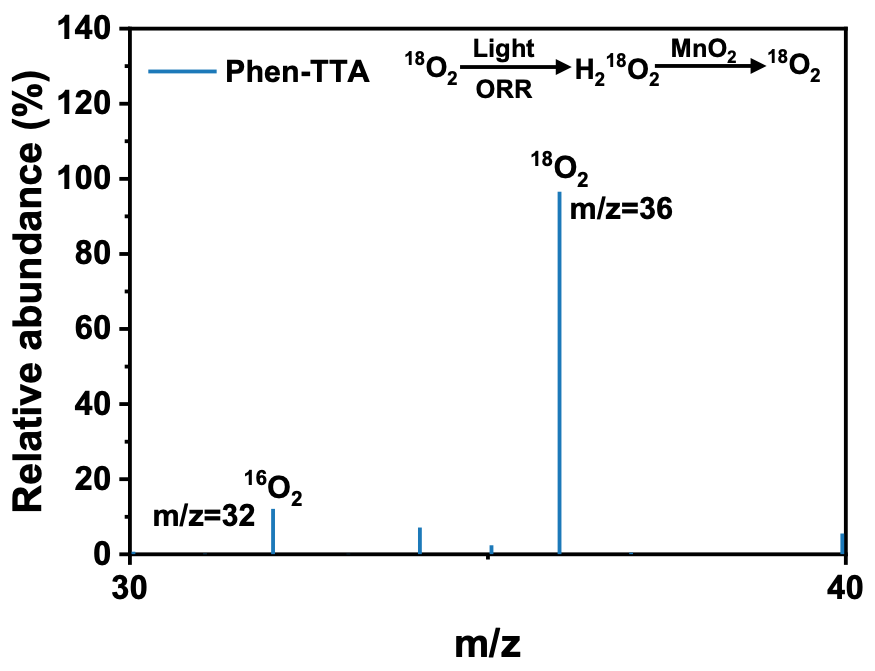


**Figure S27.** Isotopic labeling experiment with ^18^O_2_ over Phen-TTA.


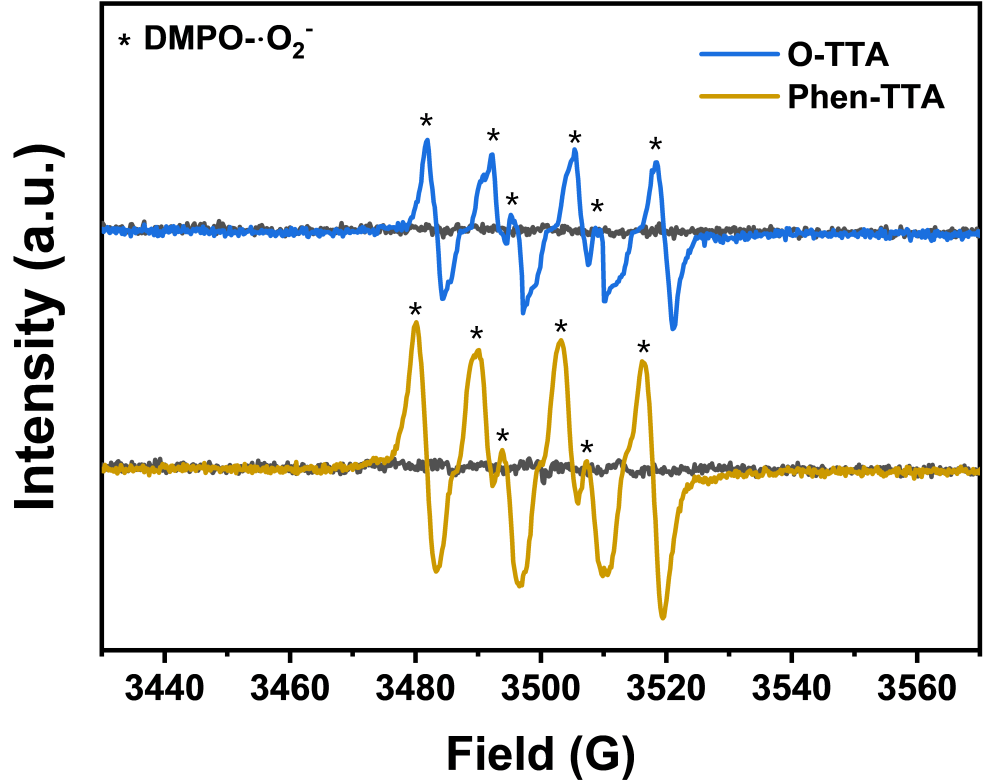


**Figure S28.** EPR spectra of COFs in methanol under the dark and visible light irradiation (DMPO- ^•^O_2_^-^).


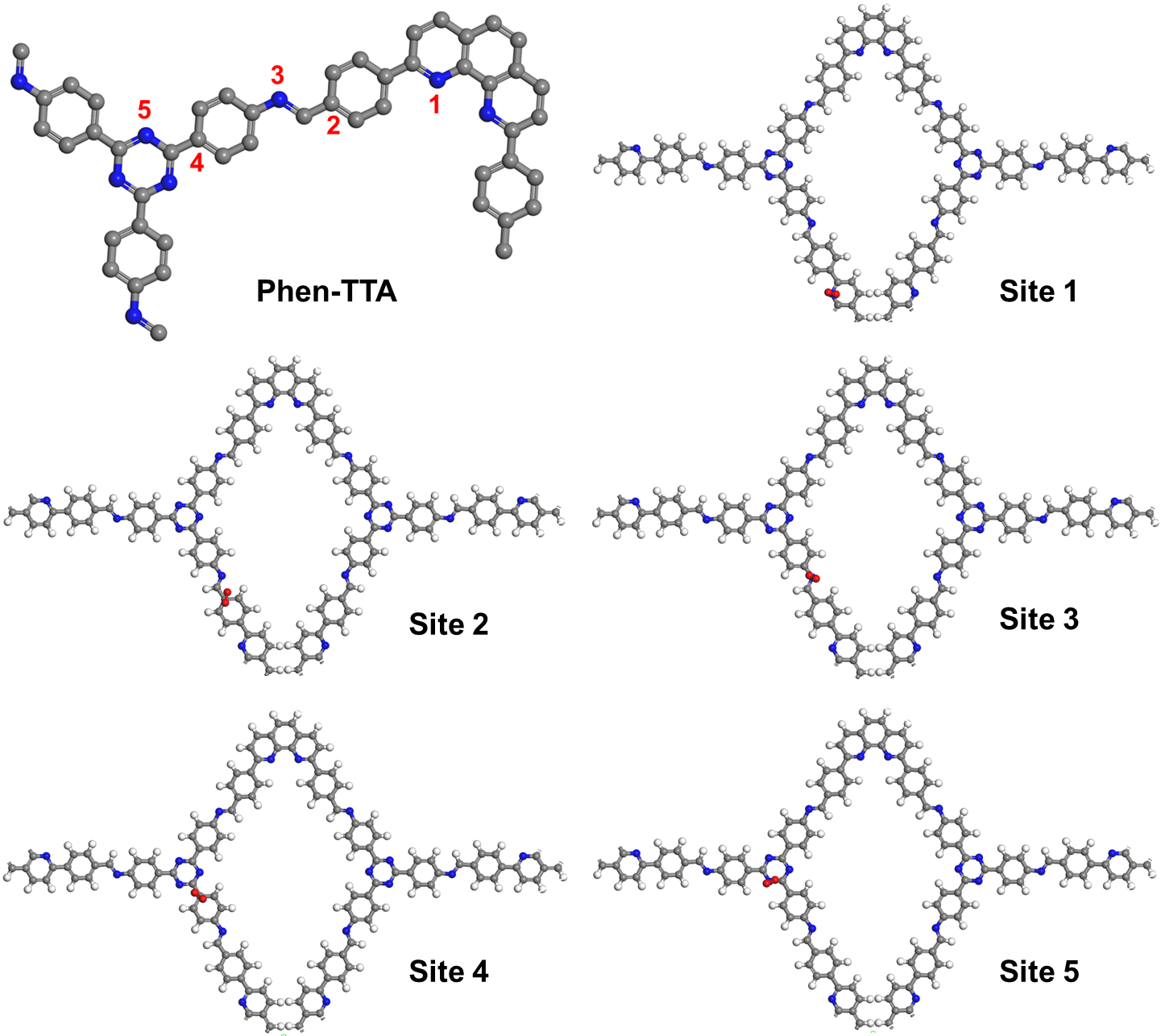


**Figure S29.** The adsorbent *O_2_ intermediates on five possible active sites on Phen-TTA. The black, white, blue, red balls represent carbon, hydrogen, nitrogen and oxygen atoms.


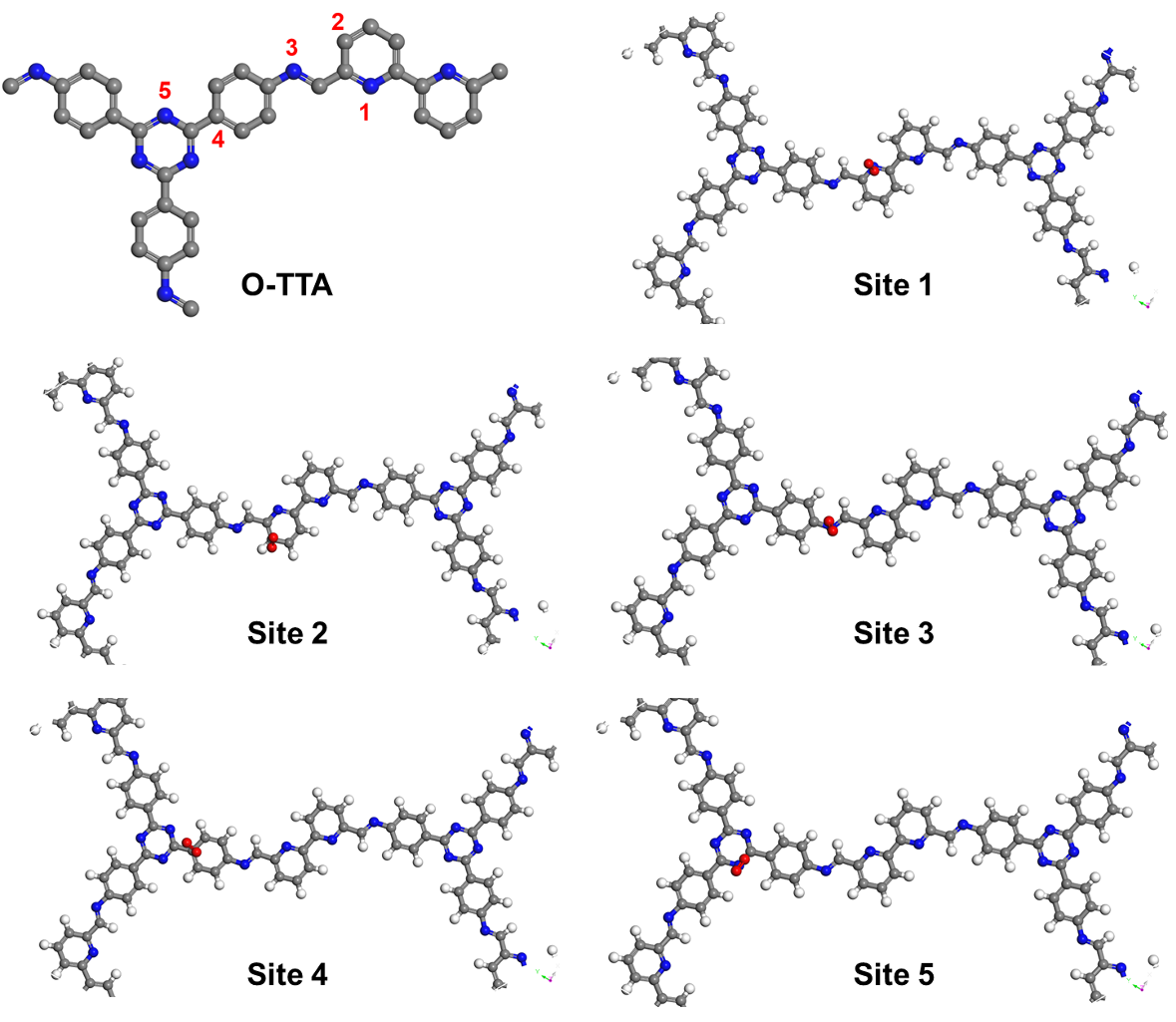


**Figure S30.** The adsorbent *O_2_ intermediates on five possible active sites on O-TTA. The black, white, blue, red balls represent carbon, hydrogen, nitrogen and oxygen atoms.


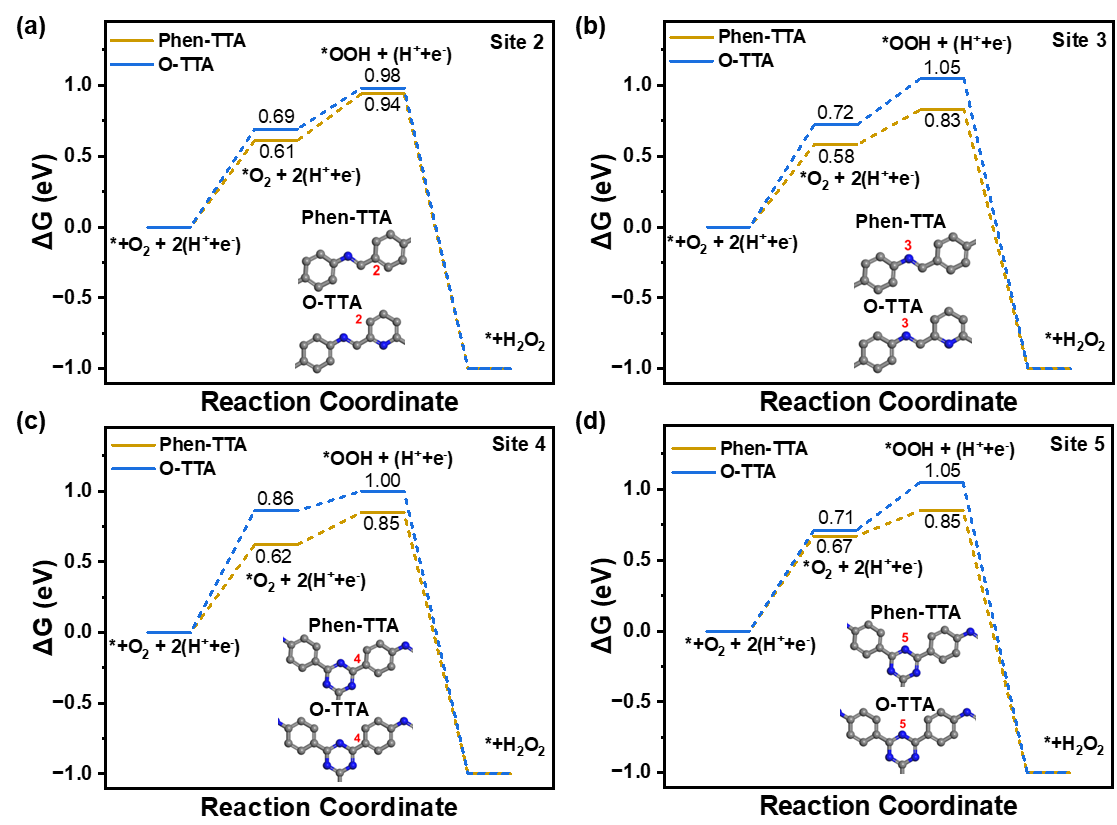


**Figure S31.** DFT calculated free energy diagrams of oxygen reduction pathways toward H_2_O_2_ generation on different active sites in O-TTA and Phen-TTA.


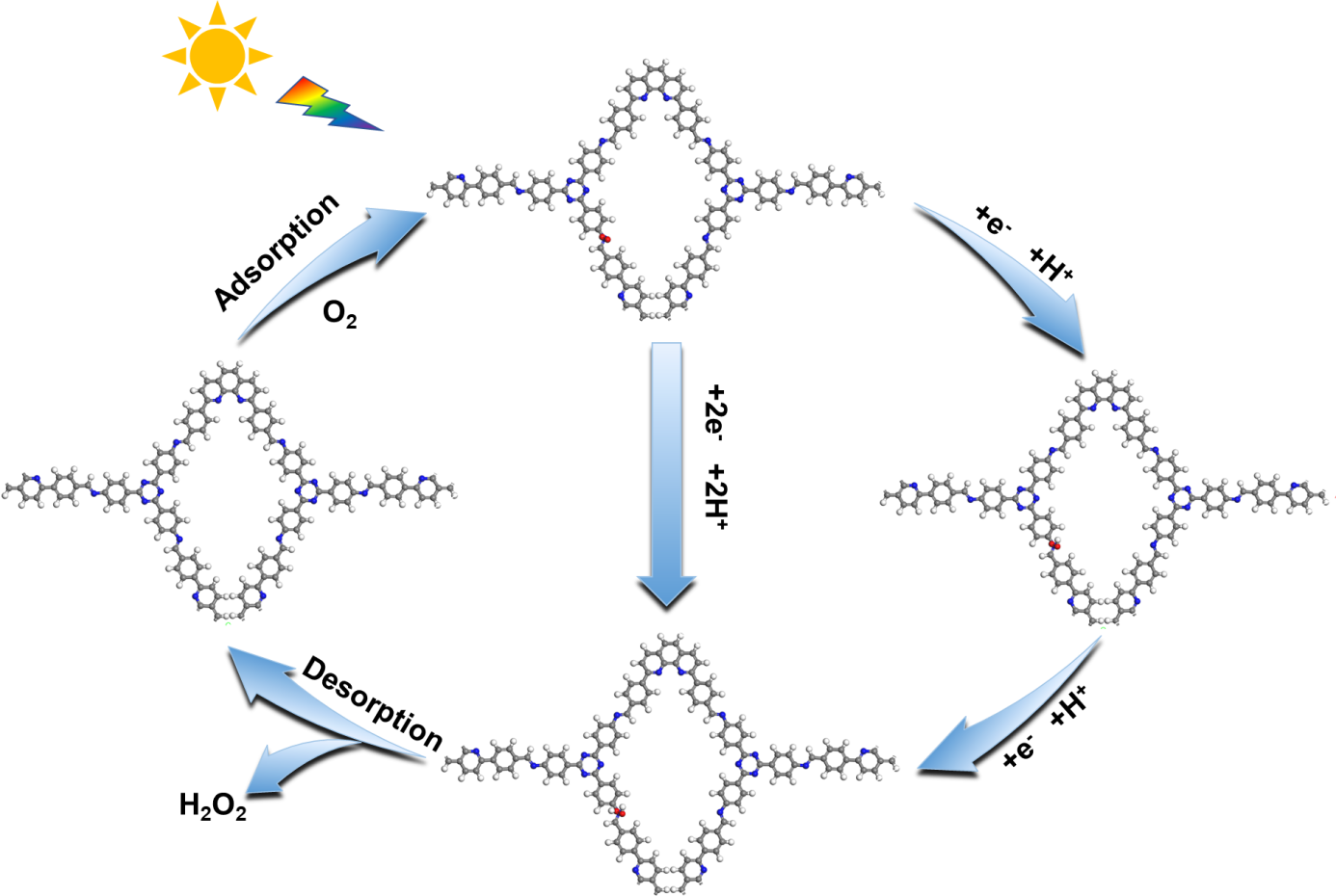


**Figure S32.** Proposed mechanism for the photosynthesis of H_2_O_2_ using Phen-TTA as the catalyst.


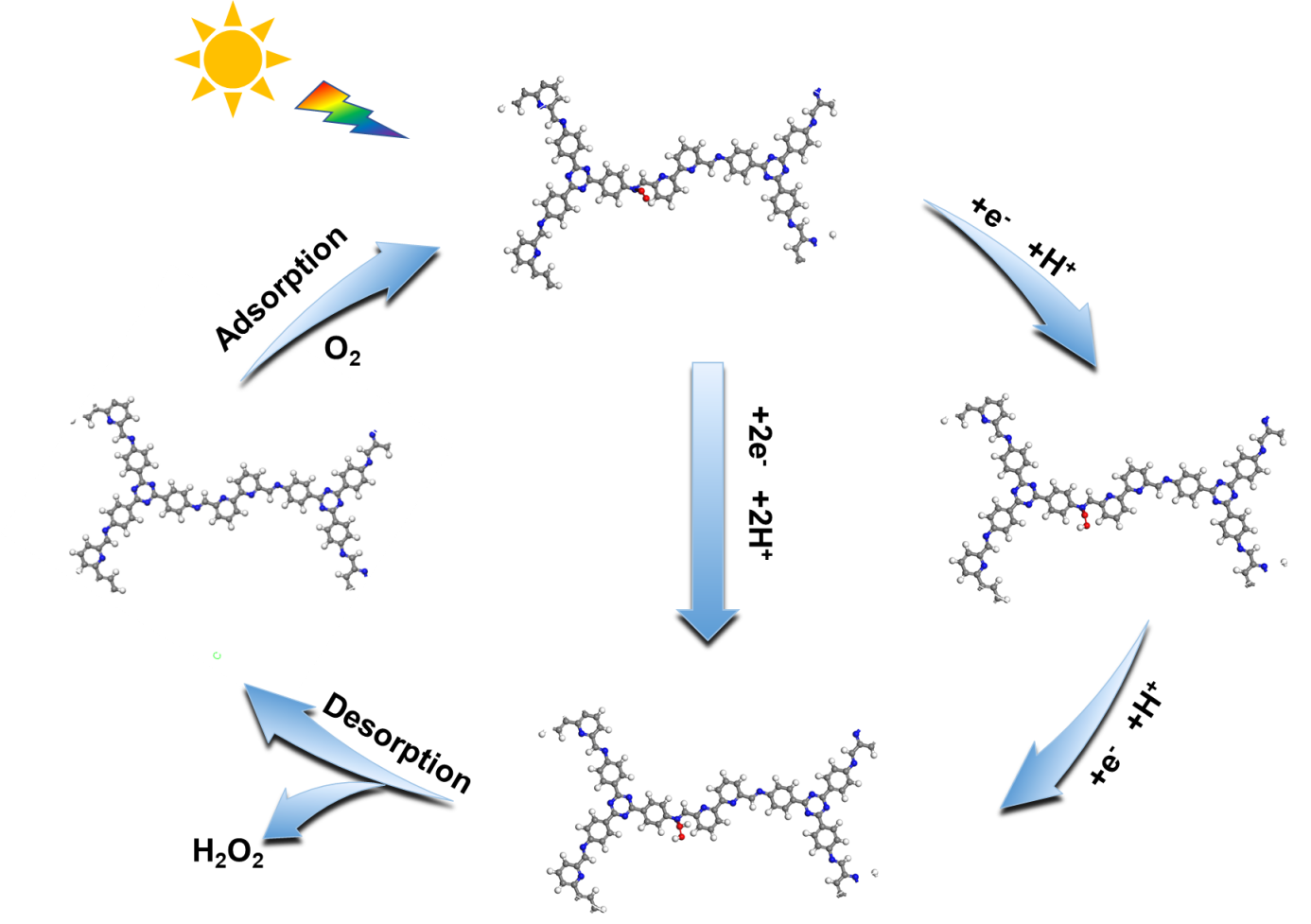


**Figure S33.** Proposed mechanism for the photosynthesis of H_2_O_2_ using O-TTA as the catalyst.


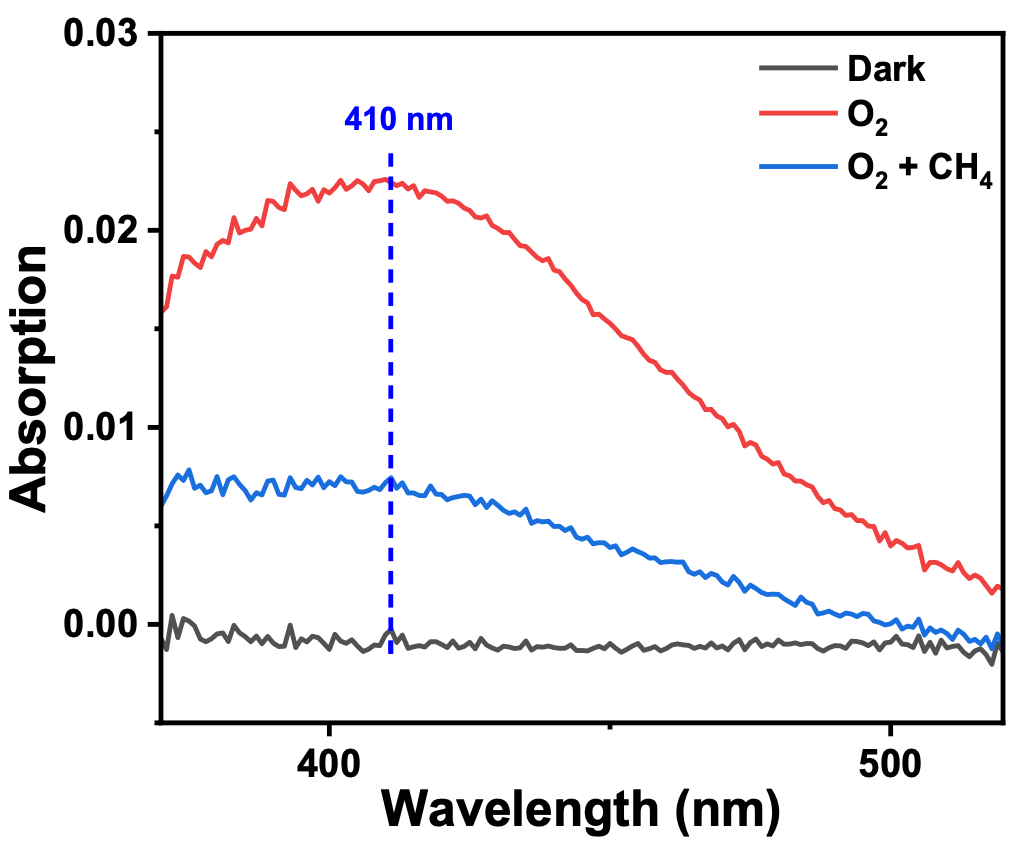


**Figure S34.** Semi-quantitatively analysis of H_2_O_2_ concentration during the photocatalytic oxidation of methane under different conditions.

Notes: in our initial experiments, no obvious H_2_O_2_ signal was detected under CH_4_/O_2_ flow. We attribute this to (i) the instability and partial decomposition of H_2_O_2_ during collection using a water bubbler under continuous gas flow, and (ii) the intrinsically low H_2_O_2_ yield under these conditions. To address these issues, we optimized the trapping method by directly bubbling the effluent gas into a titanium oxysulfate (TiOSO_4_) solution, which stabilizes H_2_O_2_ via complexation and minimizes decomposition. In addition, the reaction time was extended to 15 h to increase the accumulated concentration. With these improvements, H_2_O_2_ signals were successfully detected under both pure O_2_ and CH_4_/O_2_ atmospheres.


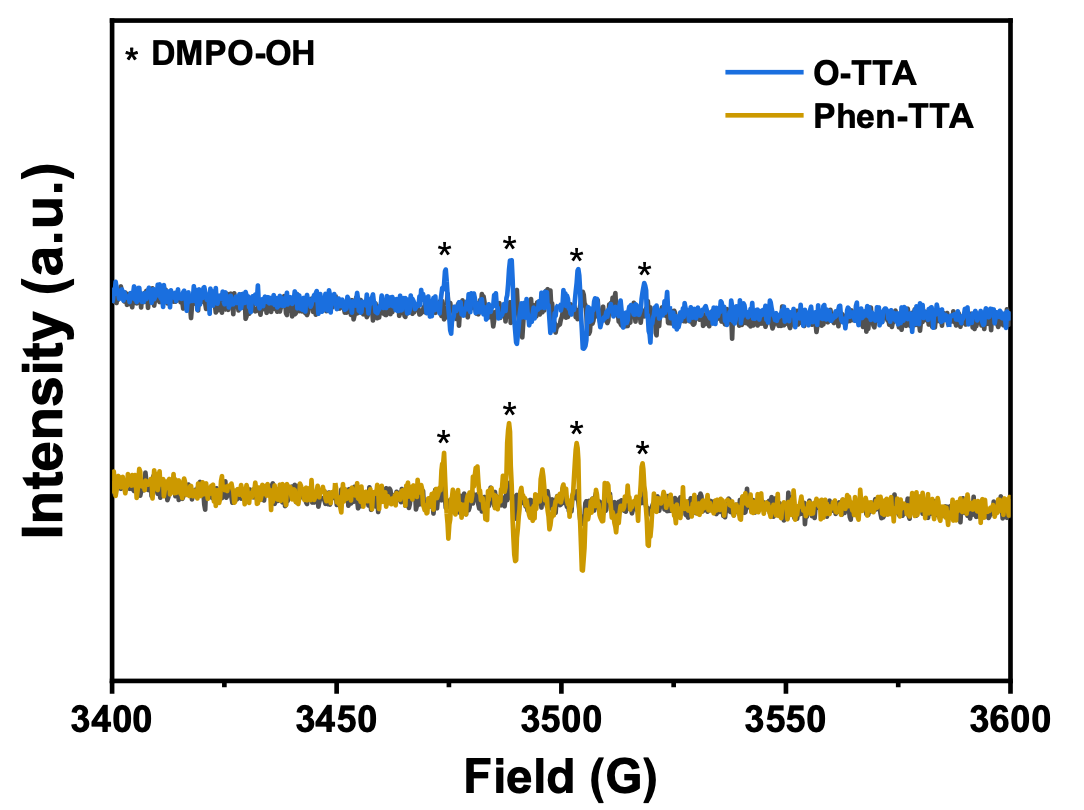


**Figure S35.** EPR spectra of COFs in water under the dark and visible light irradiation (DMPO-OH).


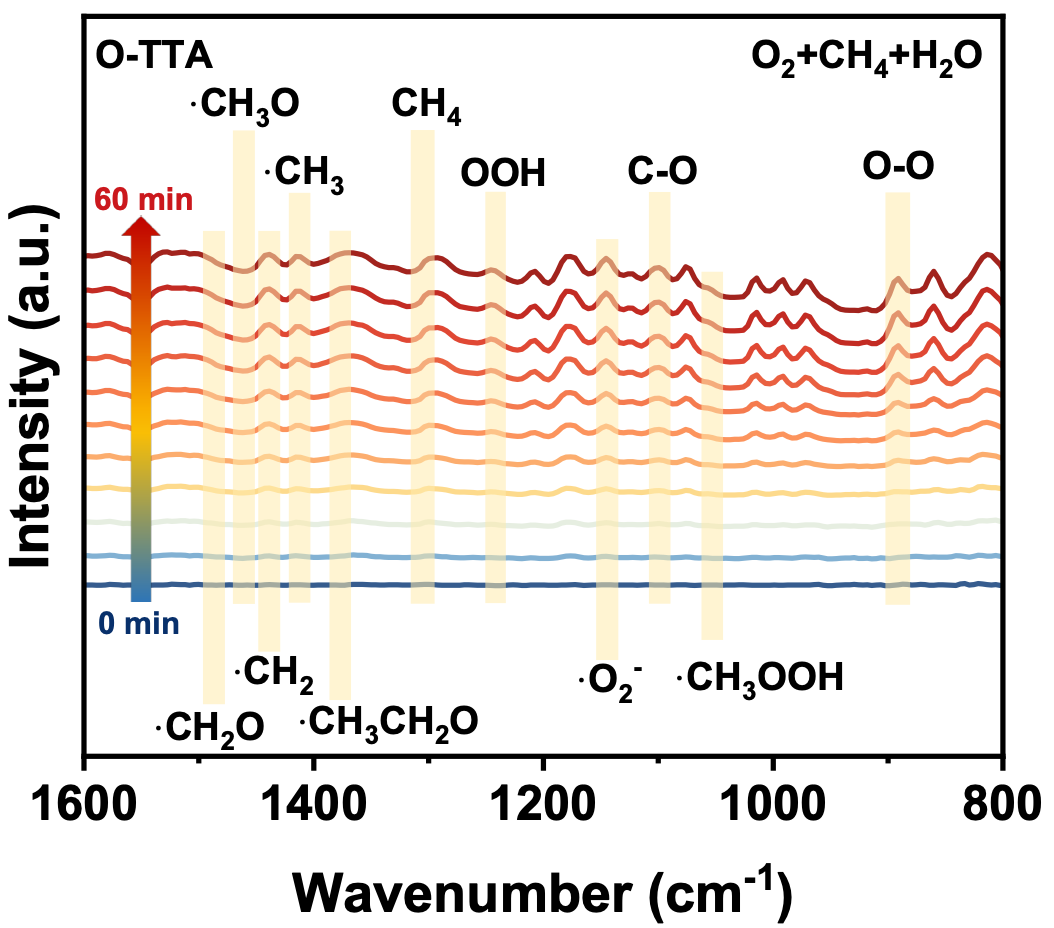


**Figure S36.** *In-situ* DRIFTS spectrum of photocatalytic CH_4_ oxidation over O-TTA during light irradiation (420 nm LED) from 0 to 60 min.


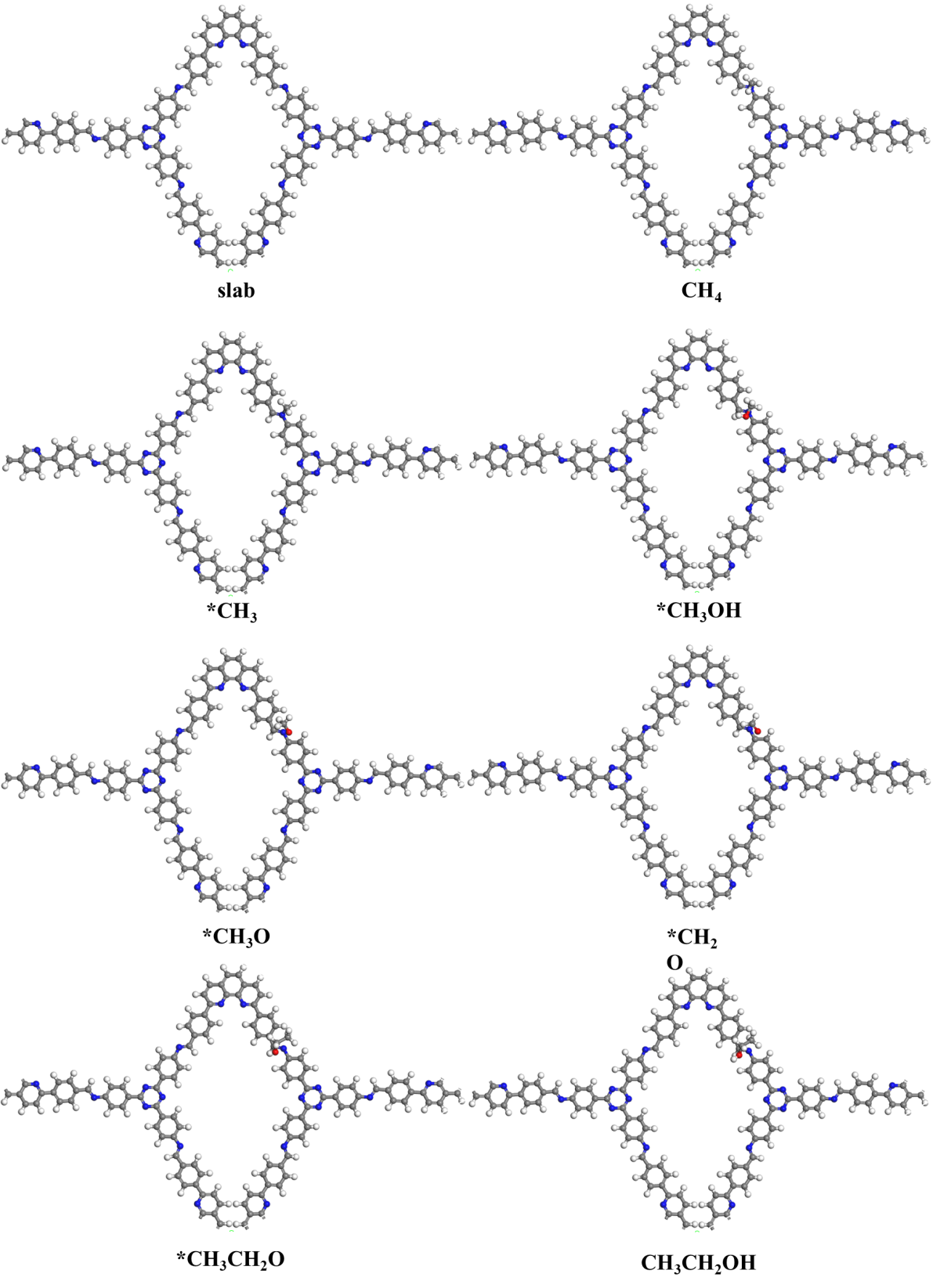


**Figure S37.** Optimized geometric structures of intermediates generated during CH_4_ oxidation over Phen-TTA.

**Section 4. Supporting Tables.**

**Table S1.** A comparison in photocatalytic H_2_O_2_ generation performance among reported COF photocatalysts and our cases.

| Photocatalyst | COF dosage | Light | H_2_O_2_  production rate  （μmol h^-1^ g_cat_^-1^） | | | Solvent(s) | AQE (%) | Reference |
| --- | --- | --- | --- | --- | --- | --- | --- | --- |
| Phen-TTA | 10 mg | 420 nm LED | | 18619 | H_2_O: BA  (9/1, 20 mL) | | 5.45  (450 nm) | This work |
| O-TTA |  |  |  | 4722 |  |  | \ |  |
| N_0_-COF | 10 mg | 495 nm LED | | 1570 | H_2_O  (20 mL) | | \ | [5] |
| COF-TfpBpy | 5 mg | Xe lamp | | 2084 | H_2_O  (10 mL) | | ＞8  （420-550 nm） | [6] |
| TF_50_-COF | 5 mg | λ> 400 nm  (300 W Xe lamp) | | 1739 | H_2_O: EtOH  (9/1, 50 mL) | | 5.1  （400 nm） | [7] |
| TD-COF | 1 mg | 400-700 nm  (white LED) | | 4620 | H_2_O  (4 mL) | | \ | [8] |
| TZ-COF | 10 mg | 420 nm LED | | 5710 | H_2_O: BA  (9/1, 20 mL) | | \ | [9] |
| PMCR-1 | 10 mg | λ> 420 nm  (300 W Xe lamp) | | 5500 | H_2_O: BA  (10/1, 22 mL) | | 14  (420 nm) | [10] |
| NiPc-THHI-COF | 5 mg | λ> 400 nm  (300 W Xe lamp) | | 4589 | H_2_O  (50 mL) | | \ | [11] |
| TACOF-1-COOH | 2.5 mg | λ> 420 nm  (300 W Xe lamp) | | 3542 | H_2_O  (18 mL) | | 5.7  (420 nm) | [12] |
| *o*-COF-TpPzda | 5 mg | λ> 420 nm  (300 W Xe lamp) | | 4396 | H_2_O  (40 mL) | | \ | [13] |
| COF-JLU90 | 5 mg | λ> 420 nm  (300 W Xe lamp) | | 9800 | H_2_O  (50 mL) | | 8.7  (450 nm) | [14] |
| Pylm-BT-F | 10 mg | λ> 420 nm  (300 W Xe lamp) | | 5342 | H_2_O: IPA  (9/1, 50 mL) | | 2.7  (420 nm) | [15] |
| MeO-QN-TA-COF | 4 mg | 460 nm LED | | 7384 | H_2_O  (20 mL) | | 10.07  (460 nm) | [16] |
| DVA-COF | 10 mg | 420nm LED | | 8450 | H_2_O: BA  (9/1, 20 mL) | | 2.84  (420 nm) | [17] |
| Tapt-Btt | 15 mg | λ>420 nm  (300 W Xe lamp) | | 140733 | H_2_O  (10 mL) | | 4.6  (450 nm) | [18] |
| sonoCOF-F2 | 50 mg | λ>420 nm  (300 W Xe lamp) | | 414.6 | H2O: BA (1/9, 30  mL) | | 4.8  (420 nm) | [19] |
| Bpy-COF | 10 mg | 420nm LED | | 2570 | H_2_O: BA  (9/1, 20 mL) | | 1.32  (420 nm) | [20] |
| TMT-NA | 3 mg | λ>400 nm  (300 W Xe lamp) | | 1593 | H_2_O  (20 mL) | | \ | [21] |
| TP-PZ-COF | 5 mg | λ>420 nm  (300 W Xe lamp) | | 4961 | H_2_O  (50 mL) | | 2.53  (600 nm) | [22] |
| OPV-TAPT-COF | 3 mg | λ>420 nm  (300 W Xe lamp) | | 3671 | H_2_O  (30 mL) | | 1.10  (420 nm) | [23] |
| TP-4,6-Pm | 1 mg | λ>420 nm  (300 W Xe lamp) | | 5481 | H_2_O  (5 mL) | | 10.5  (480 nm) | [24] |
| TAPT-BT COF | 5 mg | λ=415 nm  (100 W LED) | | 2472 | H_2_O: BA  (9/1, 20 mL) | | \ | [25] |
| *o*BPY-COF | 10 mg | λ>420 nm  (300 W Xe lamp) | | 3638 | H_2_O  (50 mL) | | 10.5  (420 nm) | [26] |
| FMP-COF | 4 mg | λ≥420 nm  LED | | 5384 | H_2_O  (20 mL) | | 4.62  (420 nm) | [27] |
| NT-Py-COF | 2 mg | λ>420 nm  (300 W Xe lamp) | | 2546 | H_2_O: BA (4/1, 10  mL) | | 1.69  (450 nm) | [28] |

**Table S2.** Comparison of methane oxidation performance between reported organic photocatalysts and COF photocatalyst in this study.

| Photocatalyst | | | Light source | Oxidant | Product yield, μmol h^-1^ g^-1^ | | | | Sel (%) | Ref |
| --- | --- | --- | --- | --- | --- | --- | --- | --- | --- | --- |
|  |  |  |  |  | C_2_H_5_OH | CH_3_COOH | CH_3_CHO | CH_3_OH |  |  |
| g-C_3_N_4_ | | LED (365 nm) | O_2_ | 29.2 | - | - | 57.3 | 46.1 | [29] |  |
| CTF | CTF-1 |  |  | 122.4 | - | - | - | 78.6 |  |  |
|  | 3 wt% PtO_x_/CTF-1 |  |  | 167.6 | - | - | - | 79.6 |  |  |
|  | 3 wt% RuO_x_/CTF-1 |  |  | 99.2 | - | - | - | 72.2 |  |  |
| Cu_9_S_5_/Cu-CCN | | 300 W Xe lamp (full spectrum) | O_2_ | 549.7 | - | - | - | 94.8 | [30] |  |
| Phen-TTA | | LED (365 nm) | In-situ generated H_2_O_2_ | 30.1 | 7.3 | 2.0 | 2.7 | 65 | This work |  |

**Table S3.** The optimized unit cell parameters of the Phen-TTA.

| *a*=34.40 Å, *b*=34.32 Å, *c*=21.12 Å, *α*=90.00°, *β*=89.40°, *γ*=120.04° | | | |
| --- | --- | --- | --- |
| Atom | X | Y | Z |
| H1 | 0.227348 | 0.557617 | 0.493118 |
| H2 | 0.186153 | 0.474696 | 0.494764 |
| H3 | 0.311668 | 0.47522 | 0.483948 |
| H4 | 0.353144 | 0.559832 | 0.482098 |
| H5 | 0.436704 | 0.647673 | 0.479016 |
| H6 | 0.519377 | 0.688876 | 0.473296 |
| H7 | 0.519821 | 0.814748 | 0.475098 |
| H8 | 0.435454 | 0.772144 | 0.480796 |
| H9 | 0.347562 | 0.767909 | 0.496275 |
| H10 | 0.306561 | 0.809445 | 0.505978 |
| H11 | 0.180984 | 0.683865 | 0.508699 |
| H12 | 0.223369 | 0.642038 | 0.4987 |
| H13 | 0.060137 | 0.031291 | 0.603092 |
| H14 | 0.017992 | 0.94856 | 0.58629 |
| H15 | 0.214031 | 0.144233 | 0.59445 |
| H16 | 0.131315 | 0.102747 | 0.603859 |
| H17 | 0.018148 | 0.878248 | 0.568793 |
| H18 | 0.05983 | 0.838125 | 0.548536 |
| H19 | 0.094299 | 0.808361 | 0.536622 |
| H20 | 0.139203 | 0.771167 | 0.524529 |
| H21 | 0.26059 | 0.899629 | 0.529802 |
| H22 | 0.215401 | 0.937934 | 0.541916 |
| H23 | 0.266933 | 0.835405 | 0.519456 |
| H24 | 0.992094 | 0.935851 | 0.454803 |
| H25 | 0.950032 | 0.976451 | 0.438564 |
| H26 | 0.95016 | 0.783507 | 0.492927 |
| H27 | 0.991858 | 0.865381 | 0.472178 |
| H28 | 0.879029 | 0.97671 | 0.438607 |
| H29 | 0.796368 | 0.935486 | 0.44901 |
| H30 | 0.731751 | 0.901737 | 0.46324 |
| H31 | 0.649232 | 0.856594 | 0.464925 |
| H32 | 0.656179 | 0.735186 | 0.475146 |
| H33 | 0.740011 | 0.780559 | 0.473487 |
| H34 | 0.794643 | 0.727359 | 0.501222 |
| H35 | 0.74953 | 0.643748 | 0.513815 |
| H36 | 0.870974 | 0.637151 | 0.518265 |
| H37 | 0.915805 | 0.719357 | 0.505715 |
| H38 | 0.585423 | 0.728821 | 0.476667 |
| H39 | 0.743289 | 0.573171 | 0.524345 |
| H40 | 0.964329 | 0.002718 | 0.537968 |
| H41 | 0.044683 | 0.042935 | 0.504937 |
| H42 | 0.852265 | 0.044626 | 0.552015 |
| H43 | 0.893676 | 0.003032 | 0.553259 |
| H44 | 0.115491 | 0.114028 | 0.489929 |
| H45 | 0.15713 | 0.197178 | 0.491218 |
| H46 | 0.951276 | 0.200053 | 0.534215 |
| H47 | 0.913448 | 0.246021 | 0.540057 |
| H48 | 0.784775 | 0.125024 | 0.552262 |
| H49 | 0.821627 | 0.079362 | 0.546757 |
| H50 | 0.849624 | 0.253005 | 0.546937 |
| H51 | 0.575244 | 0.341438 | 0.564212 |
| H52 | 0.490881 | 0.299785 | 0.570039 |
| H53 | 0.490571 | 0.173981 | 0.571811 |
| H54 | 0.573256 | 0.21535 | 0.565986 |
| H55 | 0.656937 | 0.211167 | 0.562345 |
| H56 | 0.698285 | 0.168047 | 0.560198 |
| H57 | 0.823907 | 0.293326 | 0.548999 |
| H58 | 0.782834 | 0.335041 | 0.550918 |
| H59 | 0.787017 | 0.423658 | 0.545184 |
| H60 | 0.829365 | 0.507985 | 0.534772 |
| H61 | 0.703778 | 0.507559 | 0.538096 |
| H62 | 0.662817 | 0.424912 | 0.548217 |
| H63 | 0.187945 | 0.262706 | 0.49664 |
| H64 | 0.224988 | 0.345363 | 0.491291 |
| H65 | 0.096457 | 0.3376 | 0.503267 |
| H66 | 0.05844 | 0.253662 | 0.508954 |
| H67 | 0.160325 | 0.408508 | 0.496682 |
| H68 | 0.269831 | 0.045346 | 0.570681 |
| H69 | 0.35364 | 0.08366 | 0.569755 |
| H70 | 0.361357 | 0.212397 | 0.579809 |
| H71 | 0.278845 | 0.175156 | 0.580834 |
| H72 | 0.42456 | 0.148006 | 0.568598 |
| C1 | 0.316303 | 0.613668 | 0.487413 |
| C2 | 0.381285 | 0.681213 | 0.484108 |
| C3 | 0.314024 | 0.678782 | 0.491662 |
| C4 | 0.288228 | 0.702435 | 0.496939 |
| C5 | 0.292765 | 0.564033 | 0.487586 |
| C6 | 0.430782 | 0.707144 | 0.480443 |
| C7 | 0.245543 | 0.539204 | 0.491044 |
| C8 | 0.222698 | 0.49258 | 0.491961 |
| C9 | 0.246392 | 0.468863 | 0.489533 |
| C10 | 0.293649 | 0.493838 | 0.485822 |
| C11 | 0.316555 | 0.540613 | 0.484836 |
| C12 | 0.455274 | 0.68436 | 0.478224 |
| C13 | 0.501765 | 0.707843 | 0.475091 |
| C14 | 0.525709 | 0.755348 | 0.474148 |
| C15 | 0.501071 | 0.778039 | 0.476084 |
| C16 | 0.454408 | 0.754458 | 0.479267 |
| C17 | 0.310982 | 0.749775 | 0.499027 |
| C18 | 0.287615 | 0.772813 | 0.504586 |
| C19 | 0.240256 | 0.74921 | 0.508387 |
| C20 | 0.217587 | 0.701832 | 0.505944 |
| C21 | 0.241047 | 0.678721 | 0.500401 |
| C22 | 0.07806 | 0.944009 | 0.570185 |
| C23 | 0.126081 | 0.966845 | 0.564876 |
| C24 | 0.150875 | 0.015908 | 0.572967 |
| C25 | 0.125494 | 0.037767 | 0.586339 |
| C26 | 0.077698 | 0.013053 | 0.591878 |
| C27 | 0.054603 | 0.967664 | 0.583098 |
| C28 | 0.218385 | 0.083647 | 0.577276 |
| C29 | 0.195521 | 0.107868 | 0.588669 |
| C30 | 0.149505 | 0.084896 | 0.593837 |
| C31 | 0.054665 | 0.89681 | 0.56404 |
| C32 | 0.077945 | 0.874598 | 0.552954 |
| C33 | 0.125567 | 0.899525 | 0.548793 |
| C34 | 0.151812 | 0.87639 | 0.539944 |
| C35 | 0.130786 | 0.829085 | 0.534908 |
| C36 | 0.155632 | 0.807666 | 0.528112 |
| C37 | 0.202842 | 0.832895 | 0.526096 |
| C38 | 0.223957 | 0.88001 | 0.531101 |
| C39 | 0.199042 | 0.901476 | 0.537828 |
| C40 | 0.230287 | 0.811762 | 0.519523 |
| C41 | 0.884701 | 0.917485 | 0.456018 |
| C42 | 0.859272 | 0.870203 | 0.46969 |
| C43 | 0.884004 | 0.845921 | 0.477478 |
| C44 | 0.932004 | 0.871162 | 0.471579 |
| C45 | 0.955502 | 0.918314 | 0.458415 |
| C46 | 0.932463 | 0.940615 | 0.449922 |
| C47 | 0.884481 | 0.779094 | 0.493553 |
| C48 | 0.932077 | 0.801852 | 0.488777 |
| C49 | 0.955366 | 0.847382 | 0.477403 |
| C50 | 0.860781 | 0.940636 | 0.44882 |
| C51 | 0.814798 | 0.917594 | 0.454554 |
| C52 | 0.791857 | 0.870462 | 0.466199 |
| C53 | 0.742145 | 0.844186 | 0.468446 |
| C54 | 0.858261 | 0.729652 | 0.502735 |
| C55 | 0.715827 | 0.865187 | 0.46588 |
| C56 | 0.669352 | 0.84022 | 0.46685 |
| C57 | 0.647218 | 0.792956 | 0.470529 |
| C58 | 0.673272 | 0.771871 | 0.472769 |
| C59 | 0.719853 | 0.796894 | 0.471849 |
| C60 | 0.811036 | 0.707341 | 0.505238 |
| C61 | 0.78616 | 0.66089 | 0.512236 |
| C62 | 0.807316 | 0.634992 | 0.517147 |
| C63 | 0.854519 | 0.657145 | 0.514763 |
| C64 | 0.879323 | 0.703471 | 0.507696 |
| C65 | 0.598466 | 0.765457 | 0.472859 |
| C66 | 0.779942 | 0.586339 | 0.524003 |
| C67 | 0.050902 | 0.109023 | 0.507484 |
| C68 | 0.029084 | 0.134947 | 0.515942 |
| C69 | 0.980319 | 0.110548 | 0.527346 |
| C70 | 0.958363 | 0.062718 | 0.535719 |
| C71 | 0.982414 | 0.039003 | 0.530044 |
| C72 | 0.026711 | 0.06117 | 0.513018 |
| C73 | 0.912848 | 0.110239 | 0.538125 |
| C74 | 0.888615 | 0.062731 | 0.546033 |
| C75 | 0.911502 | 0.039465 | 0.54623 |
| C76 | 0.097765 | 0.132673 | 0.496989 |
| C77 | 0.120778 | 0.178905 | 0.497213 |
| C78 | 0.096669 | 0.202223 | 0.505162 |
| C79 | 0.889611 | 0.136693 | 0.54018 |
| C80 | 0.914865 | 0.184005 | 0.5381 |
| C81 | 0.89366 | 0.209321 | 0.541284 |
| C82 | 0.846617 | 0.188486 | 0.546341 |
| C83 | 0.8212 | 0.141185 | 0.548249 |
| C84 | 0.842387 | 0.115927 | 0.545206 |
| C85 | 0.825792 | 0.216357 | 0.548938 |
| C86 | 0.629022 | 0.304501 | 0.560667 |
| C87 | 0.6939 | 0.301974 | 0.556998 |
| C88 | 0.696348 | 0.369455 | 0.552759 |
| C89 | 0.722159 | 0.418978 | 0.54728 |
| C90 | 0.579539 | 0.280925 | 0.564539 |
| C91 | 0.717372 | 0.275889 | 0.556678 |
| C92 | 0.556126 | 0.304745 | 0.565782 |
| C93 | 0.509474 | 0.281729 | 0.569033 |
| C94 | 0.484634 | 0.234328 | 0.570967 |
| C95 | 0.50836 | 0.210623 | 0.57001 |
| C96 | 0.554853 | 0.233574 | 0.566819 |
| C97 | 0.693519 | 0.228604 | 0.559459 |
| C98 | 0.716354 | 0.204742 | 0.558302 |
| C99 | 0.763601 | 0.227114 | 0.554387 |
| C100 | 0.787365 | 0.274602 | 0.551945 |
| C101 | 0.76459 | 0.298372 | 0.553021 |
| C102 | 0.769328 | 0.442595 | 0.543527 |
| C103 | 0.792769 | 0.489232 | 0.537747 |
| C104 | 0.770081 | 0.513849 | 0.535351 |
| C105 | 0.722732 | 0.489941 | 0.539447 |
| C106 | 0.699389 | 0.443477 | 0.545239 |
| C107 | 0.120021 | 0.251999 | 0.503121 |
| C108 | 0.167258 | 0.278524 | 0.498179 |
| C109 | 0.188552 | 0.325052 | 0.495217 |
| C110 | 0.163226 | 0.346951 | 0.497114 |
| C111 | 0.116171 | 0.320673 | 0.502058 |
| C112 | 0.094861 | 0.274074 | 0.505159 |
| C113 | 0.184126 | 0.395731 | 0.494661 |
| C114 | 0.268089 | 0.107045 | 0.575587 |
| C115 | 0.290155 | 0.081892 | 0.572461 |
| C116 | 0.33672 | 0.103367 | 0.571937 |
| C117 | 0.36298 | 0.15057 | 0.574304 |
| C118 | 0.341072 | 0.175852 | 0.577773 |
| C119 | 0.294605 | 0.154425 | 0.578361 |
| C120 | 0.411709 | 0.17172 | 0.57223 |
| N1 | 0.291064 | 0.633519 | 0.49136 |
| N2 | 0.361414 | 0.635988 | 0.483744 |
| N3 | 0.359117 | 0.704164 | 0.487945 |
| N4 | 0.19616 | 0.038914 | 0.569247 |
| N5 | 0.14848 | 0.944446 | 0.554453 |
| N6 | 0.213125 | 0.768804 | 0.51446 |
| N7 | 0.861591 | 0.801074 | 0.488175 |
| N8 | 0.814001 | 0.847917 | 0.473943 |
| N9 | 0.572575 | 0.782573 | 0.471288 |
| N10 | 0.957543 | 0.13307 | 0.529562 |
| N11 | 0.051982 | 0.180318 | 0.513776 |
| N12 | 0.651277 | 0.349686 | 0.556752 |
| N13 | 0.648791 | 0.279118 | 0.560895 |
| N14 | 0.719219 | 0.347139 | 0.552922 |
| N15 | 0.797171 | 0.560652 | 0.529005 |
| N16 | 0.782876 | 0.199535 | 0.552836 |
| N17 | 0.227049 | 0.421934 | 0.490903 |
| N18 | 0.437797 | 0.214786 | 0.573756 |

**Table S4.** The optimized unit cell parameters of the O-TTA.

| *a*=40.54 Å, *b*=41.10 Å, *c*=14.98 Å, *α*=89.95°, *β*=90.54°, *γ*=119.34° | | | |
| --- | --- | --- | --- |
| Atom | X | Y | Z |
| H1 | 0.473076 | 0.717125 | 0.466812 |
| H2 | 0.521543 | 0.785115 | 0.461684 |
| H3 | 0.43184 | 0.812922 | 0.463177 |
| H4 | 0.384597 | 0.74598 | 0.468684 |
| H5 | 0.337079 | 0.551172 | 0.476893 |
| H6 | 0.358097 | 0.502934 | 0.477492 |
| H7 | 0.474331 | 0.590488 | 0.470597 |
| H8 | 0.453358 | 0.637636 | 0.470081 |
| H9 | 0.30283 | 0.685991 | 0.473988 |
| H10 | 0.233245 | 0.664859 | 0.478255 |
| H11 | 0.209255 | 0.550511 | 0.485381 |
| H12 | 0.277597 | 0.571655 | 0.480253 |
| H13 | 0.644271 | 0.568438 | 0.465099 |
| H14 | 0.574395 | 0.547024 | 0.468314 |
| H15 | 0.664976 | 0.519508 | 0.465764 |
| H16 | 0.411468 | 0.444273 | 0.477248 |
| H17 | 0.43219 | 0.395356 | 0.477947 |
| H18 | 0.502057 | 0.416764 | 0.474771 |
| H19 | 0.591381 | 0.41609 | 0.47156 |
| H20 | 0.485055 | 0.547693 | 0.471606 |
| H21 | 0.603352 | 0.246657 | 0.476248 |
| H22 | 0.554873 | 0.178669 | 0.481354 |
| H23 | 0.644552 | 0.150831 | 0.47985 |
| H24 | 0.691811 | 0.217776 | 0.474412 |
| H25 | 0.73936 | 0.412606 | 0.466159 |
| H26 | 0.718337 | 0.460846 | 0.465502 |
| H27 | 0.602105 | 0.373292 | 0.47234 |
| H28 | 0.623071 | 0.326143 | 0.472951 |
| H29 | 0.773614 | 0.277782 | 0.469066 |
| H30 | 0.843198 | 0.298928 | 0.464785 |
| H31 | 0.867152 | 0.413264 | 0.457555 |
| H32 | 0.798811 | 0.392101 | 0.462737 |
| H33 | 0.047964 | 0.583991 | 0.488975 |
| H34 | 0.004304 | 0.514626 | 0.478899 |
| H35 | 0.118642 | 0.608159 | 0.489516 |
| H36 | 0.156376 | 0.539507 | 0.470966 |
| H37 | 0.496492 | 0.067034 | 0.492921 |
| H38 | 0.448542 | 0.998231 | 0.493501 |
| H39 | 0.47212 | 0.951944 | 0.481457 |
| H40 | 0.601894 | 0.09856 | 0.472648 |
| H41 | 0.627829 | 0.965564 | 0.449534 |
| H42 | 0.604239 | 0.011847 | 0.461586 |
| H43 | 0.579879 | 0.89676 | 0.450109 |
| H44 | 0.474487 | 0.865204 | 0.470397 |
| H45 | 0.028469 | 0.379816 | 0.454054 |
| H46 | 0.072128 | 0.449188 | 0.464162 |
| H47 | 0.957787 | 0.355653 | 0.453534 |
| H48 | 0.920048 | 0.424285 | 0.472119 |
| C1 | 0.335297 | 0.641735 | 0.47448 |
| C2 | 0.382098 | 0.626251 | 0.473184 |
| C3 | 0.397005 | 0.687344 | 0.47071 |
| C4 | 0.42577 | 0.727284 | 0.468076 |
| C5 | 0.394014 | 0.597494 | 0.473466 |
| C6 | 0.294507 | 0.630088 | 0.476471 |
| C7 | 0.464548 | 0.738477 | 0.466147 |
| C8 | 0.491377 | 0.776111 | 0.463358 |
| C9 | 0.480465 | 0.8038 | 0.462638 |
| C10 | 0.44142 | 0.792353 | 0.464325 |
| C11 | 0.414705 | 0.754805 | 0.467168 |
| C12 | 0.367064 | 0.55938 | 0.475534 |
| C13 | 0.3786 | 0.532642 | 0.475868 |
| C14 | 0.417288 | 0.543038 | 0.474136 |
| C15 | 0.44421 | 0.581389 | 0.472017 |
| C16 | 0.432687 | 0.608018 | 0.471698 |
| C17 | 0.281931 | 0.656425 | 0.476028 |
| C18 | 0.243446 | 0.644769 | 0.478453 |
| C19 | 0.21639 | 0.606585 | 0.481114 |
| C20 | 0.229255 | 0.580254 | 0.482198 |
| C21 | 0.267584 | 0.591898 | 0.479604 |
| C22 | 0.623539 | 0.538761 | 0.466734 |
| C23 | 0.58493 | 0.527216 | 0.468481 |
| C24 | 0.558954 | 0.488811 | 0.470573 |
| C25 | 0.607376 | 0.474341 | 0.46922 |
| C26 | 0.635248 | 0.512143 | 0.467085 |
| C27 | 0.517482 | 0.474977 | 0.472522 |
| C28 | 0.469074 | 0.489446 | 0.473838 |
| C29 | 0.4412 | 0.451636 | 0.475958 |
| C30 | 0.452922 | 0.425025 | 0.476339 |
| C31 | 0.491522 | 0.43657 | 0.474607 |
| C32 | 0.616602 | 0.444032 | 0.469764 |
| C33 | 0.459844 | 0.51975 | 0.47329 |
| C34 | 0.741128 | 0.322032 | 0.468615 |
| C35 | 0.694337 | 0.337522 | 0.46992 |
| C36 | 0.679417 | 0.276421 | 0.47242 |
| C37 | 0.650647 | 0.23649 | 0.475013 |
| C38 | 0.682426 | 0.366285 | 0.469578 |
| C39 | 0.781923 | 0.333685 | 0.46655 |
| C40 | 0.61188 | 0.225311 | 0.47692 |
| C41 | 0.585039 | 0.187677 | 0.47969 |
| C42 | 0.59594 | 0.159982 | 0.480422 |
| C43 | 0.634987 | 0.171419 | 0.478731 |
| C44 | 0.661712 | 0.208975 | 0.475928 |
| C45 | 0.709375 | 0.404407 | 0.467516 |
| C46 | 0.697831 | 0.431137 | 0.467146 |
| C47 | 0.659145 | 0.420739 | 0.468839 |
| C48 | 0.632224 | 0.382385 | 0.470966 |
| C49 | 0.643753 | 0.35576 | 0.471331 |
| C50 | 0.794509 | 0.30735 | 0.467005 |
| C51 | 0.833 | 0.319023 | 0.464562 |
| C52 | 0.860043 | 0.357209 | 0.46188 |
| C53 | 0.847168 | 0.383527 | 0.460792 |
| C54 | 0.808842 | 0.371871 | 0.463402 |
| C55 | 0.059318 | 0.564825 | 0.485001 |
| C56 | 0.034924 | 0.526434 | 0.479494 |
| C57 | 0.050448 | 0.502521 | 0.474463 |
| C58 | 0.111356 | 0.552628 | 0.479685 |
| C59 | 0.098228 | 0.57848 | 0.485396 |
| C60 | 0.151648 | 0.563621 | 0.477939 |
| C61 | 0.530547 | 0.995016 | 0.475071 |
| C62 | 0.54338 | 0.056323 | 0.480979 |
| C63 | 0.504871 | 0.045608 | 0.488105 |
| C64 | 0.47874 | 0.00766 | 0.488179 |
| C65 | 0.491458 | 0.98183 | 0.481596 |
| C66 | 0.573361 | 0.095437 | 0.479079 |
| C67 | 0.597623 | 0.956124 | 0.454863 |
| C68 | 0.584903 | 0.981956 | 0.461446 |
| C69 | 0.545808 | 0.968759 | 0.467962 |
| C70 | 0.532987 | 0.907455 | 0.462057 |
| C71 | 0.571493 | 0.918177 | 0.45493 |
| C72 | 0.503021 | 0.868332 | 0.463977 |
| C73 | 0.017106 | 0.398979 | 0.45802 |
| C74 | 0.041502 | 0.437379 | 0.463548 |
| C75 | 0.025985 | 0.461297 | 0.468573 |
| C76 | 0.965073 | 0.411179 | 0.463332 |
| C77 | 0.978198 | 0.385327 | 0.457631 |
| C78 | 0.924782 | 0.400184 | 0.465089 |
| N1 | 0.360321 | 0.678496 | 0.472368 |
| N2 | 0.344724 | 0.614521 | 0.474837 |
| N3 | 0.409299 | 0.662295 | 0.471109 |
| N4 | 0.570052 | 0.462944 | 0.470932 |
| N5 | 0.506398 | 0.500855 | 0.472163 |
| N6 | 0.425521 | 0.513577 | 0.4747 |
| N7 | 0.716099 | 0.285272 | 0.470705 |
| N8 | 0.731705 | 0.349248 | 0.468208 |
| N9 | 0.667139 | 0.301475 | 0.471959 |
| N10 | 0.650928 | 0.450214 | 0.468233 |
| N11 | 0.087947 | 0.51552 | 0.474493 |
| N12 | 0.1782 | 0.597813 | 0.48317 |
| N13 | 0.555923 | 0.031531 | 0.474876 |
| N14 | 0.566397 | 0.122829 | 0.48325 |
| N15 | 0.520441 | 0.932243 | 0.468143 |
| N16 | 0.509988 | 0.840961 | 0.459818 |
| N17 | 0.988483 | 0.448289 | 0.468519 |
| N18 | 0.89823 | 0.365991 | 0.45981 |

**Section 5. References.**

[1] a) G. Kresse, J. Furthmiiller, *Comput. Mater. Sci.* **1996**, *6,* 15-50; b) G. Kresse, J. Furthmüller, *Phys. Rev. B.* **1996**, *54,* 11169−11186.

[2] S. Grimme, J. Antony, S. Ehrlich, H. Krieg, *J. Chem. Phys.* **2010**, *132*, 154104.

[3] J. K. Nùrskov, J. Rossmeisl, A. Logadottir, L. Lindqvist, J. R. Kitchin, T. Bligaard, H. JoÂnsson, *J. Phys. Chem. B* **2004**, *108,* 17886-17892.

[4] V. Wang, N. Xu, J.-C. Liu, G. Tang, W.-T. Geng, *Comput. Phys. Commun.* **2021**, *267*, 108033.

[5] S. Chai, X. Chen, X. Zhang, Y. Fang, R. S. Sprick, X. Chen, *Environ. Sci. Nano.* **2022**, *9*, 2464−2469.

[6] M. Kou, Y. Wang, Y. Xu, L. Ye, Y. Huang, B. Jia, H. Li, J. Ren, Y. Deng, J. Chen, Y. Zhou, K. Lei, L. Wang, W. Liu, H. Huang, T. Ma, *Angew. Chem., Int. Ed.* **2022**, *61*, e202200413.

[7] H. Wang, C. Yang, F. Chen, G. Zheng, Q. Han, *Angew. Chem., Int. Ed.* **2022**, *61*, e202202328.

[8] J. Y. Yue, L. P. Song, Y. F. Fan, Z. X. Pan, P. Yang, Y. Ma, Q. Xu, B. Tang, *Angew. Chem., Int. Ed.* **2023**, *62*, e202309624.

[9] H. Yu, X. Zhang, Q. Chen, P.-K. Zhou, F. Xu, H. Wang, X. Chen, *Chem. Res. Chin. Univ.* **2024**, *41*, 734−740.

[10] P. Das, J. Roeser, A. Thomas, *Angew. Chem., Int. Ed.* **2023**, *62*, e202304349.

[11] X. Wang, Y. Jin, N. Li, H. Zhang, X. Liu, X. Yang, H. Pan, T. Wang, K. Wang, D. Qi, J. Jiang, *Angew. Chem., Int. Ed.* **2024**, *63*, e202401014.

[12] H. Xu, Y. Wang, Y. Xu, Q. Wang, M. Zhuang, Q. Liao, K. Xi, *Angew. Chem., Int. Ed.* **2024**, *63*, e202408802.

[13] T. Yang, D. Zhang, A. Kong, Y. Zou, L. Yuan, C. Liu, S. Luo, G. Wei, C. Yu, *Angew. Chem., Int. Ed.* **2024**, *63*, e202404077.

[14] Z. Zhang, Y. Hou, S. Zhu, L. Yang, Y. Wang, H. Yue, H. Xia, G. Wu, S. W. Yang, X. Liu, *Angew. Chem., Int. Ed.* **2025**, *64*, e202505286.

[15] Z. Li, B. Cai, Y. Zou, D. Zhang, Y. Liang, Y. Zhou, Y. Ma, X. Wang, B. Shi, W. K. Chen, Y. Liu, X. Zhao, *Adv. Energy Mater.* **2025**, *15*, 2500341.

[16] R. Liu, M. Zhang, F. Zhang, B. Zeng, X. Li, Z. Guo, X. Lang, *Small.* **2025**, *21*, 2411625.

[17] H. Yu, F. Zhang, Q. Chen, P. K. Zhou, W. Xing, S. Wang, G. Zhang, Y. Jiang, X. Chen, *Angew. Chem., Int. Ed.* **2024**, *63*, e202402297.

[18] C. Qin, X. Wu, L. Tang, X. Chen, M. Li, Y. Mou, B. Su, S. Wang, C. Feng, J. Liu, X. Yuan, Y. Zhao, H. Wang, *Nat. Commun.* **2023**, *14*, 5238.

[19] W. Zhao, P. Yan, B. Li, M. Bahri, L. Liu, X. Zhou, R.; Clowes, N. D. Browning, Y. Wu, J. W. Ward, A. I. Cooper, *J. Am. Chem. Soc.* **2022**, *144*, 9902−9909.

[20] J. Lu, C. Lin, C. Li, H. Shi, N. Liu, W. Xing, S. Wang, G. Zhang, T.-T. Chen, X. Chen, *Chin. J. Catal.* **2026**, *81*, 185−194.

[21] T. Chen, M. Xie, W. Wu, X. Chen, L. Zhao, R.-S. Zhao, *Chem. Eng. J.* **2026**, *530*, 173571

[22] Z. Yu, J. Zhang, X. Zhang, X. Sun, G. Wu, Z. Zhang, F. Yu, J. Hua, *Chem. Sci.* **2026**, DOI: 10.1039/D5SC08298F.

[23] J. Fan, S. Lv, Y. Yan, Q. Li, S.-X. Lin, D. Yuan, *Green Chem.* **2026**, DOI: 10.1039/D6GC00046K.

[24] A. Wang, S. Pan, G. Wang, X. Song, T. Li, L. Wang, *ACS Energy Lett.* **2026**, *11*, 2235-2246.

[25] Y. Zhou, C. You, B. Hu, Y. Ma, C. Liu, *J. Alloys Compd.* **2026**, *1056*, 186640.

[26] S. Liu, C. Zhu, H. Zhang, J. Wang, Q. Fang, C. Xu, S. Song, Y. Shen, *ACS Catal.* **2025**, *16*, 1372−1385.

[27] N. Qin, L. Li, Y. Huo, J. Liu, Y. Wang, N. Huang, L. Mi, B. Zhang, *ACS Sustainable Chem. Eng.* **2025**, *14*, 738−747.

[28] H. Shi, D. Guo, H. Zhang, R. Ma, K. Chi, Y. Zhao, *Green Chem.* **2026**, DOI: 10.1039/D5GC05666G.

[29] J. Xie, C. Fu, M. G. Quesne, J. Guo, C. Wang, L. Xiong, C. D. Windle, S. Gadipelli, Z. X. Guo, W. Huang, C. R. A. Catlow, J. Tang, *Nature* **2025**, *639*, 368−374.

[30] F. Xue, C. Zhang, C. Cheng, X. Yan, F. Liu, X. Liu, B. Jiang, Q. Zhang, L. Sun, H. Peng, W.-H. Huang, C.-W. Pao, Z. Hu, M. Chen, D. Su, M. Liu, X. Huang, Y. Xu, *Nat. Commun.* **2024**, *15*, 10451.
